# Correction: A Genomic Portrait of Haplotype Diversity and Signatures of Selection in Indigenous Southern African Populations

**DOI:** 10.1371/journal.pgen.1005363

**Published:** 2015-07-17

**Authors:** The PLOS Genetics Staff

## Notice of Republication

This article was republished on June 12, 2015, to include details of the editor who handled the submission, which had been omitted due to an error in production, and to correct a misspelling of the fifth author’s name. Please download this article again to view the correct version. The originally published, uncorrected article and the republished, corrected article are provided here for reference.

## Supporting Information

S1 File Originally published, uncorrected article. (PDF)

S2 File Republished, corrected article. (PDF)

## References

[1] Chimusa ER, Meintjies A, Tchanga M, Mulder N, Seoighe C, Soodyall H, et al (2015) A Genomic Portrait of Haplotype Diversity and Signatures of Selection in Indigenous Southern African Populations. PLoS Genet 11(3): e1005052 doi:10.1371/journal.pgen.1005052 25811879 10.1371/journal.pgen.1005052 PMC4374865

