# Supplementary figures and images for: A Genomic Portrait of Haplotype Diversity and Signatures of Selection in Indigenous Southern African Populations
Source: PLoS Genet. 2015 Mar 26;11(3):e1005052. doi: 10.1371/journal.pgen.1005052 (PMC4374865; doi:10.1371/journal.pgen.1005052)

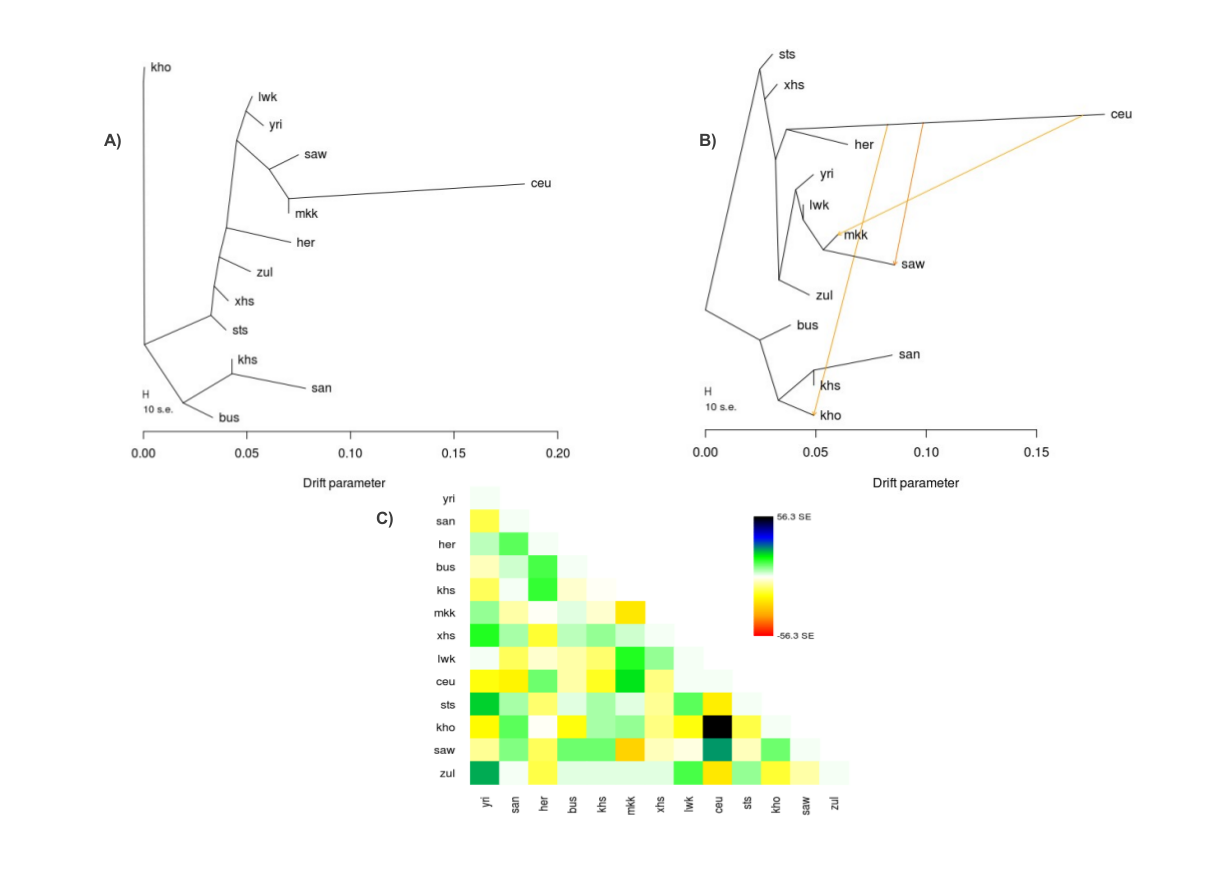

Supplement: S1 Fig — (C) Residual fit from the maximum likelihood tree is plotted and the standard error of the entries in the covariance matrix is represented ten times on the scale bar. (PNG) [file pgen.1005052.s001.png]

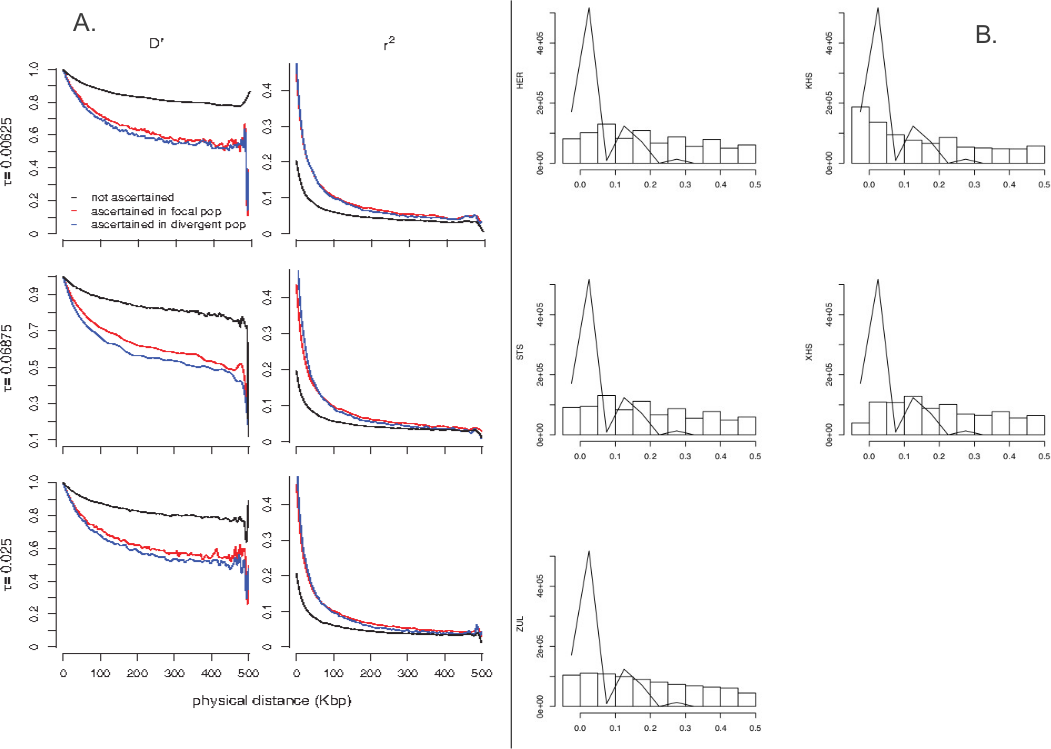

Supplement: S2 Fig — (B) Frequency spectra in the genotyped population, or in a divergent population, showing the frequency spectra to differ when SNPs are ascertained in a divergent population. (TIFF) [file pgen.1005052.s002.tiff]

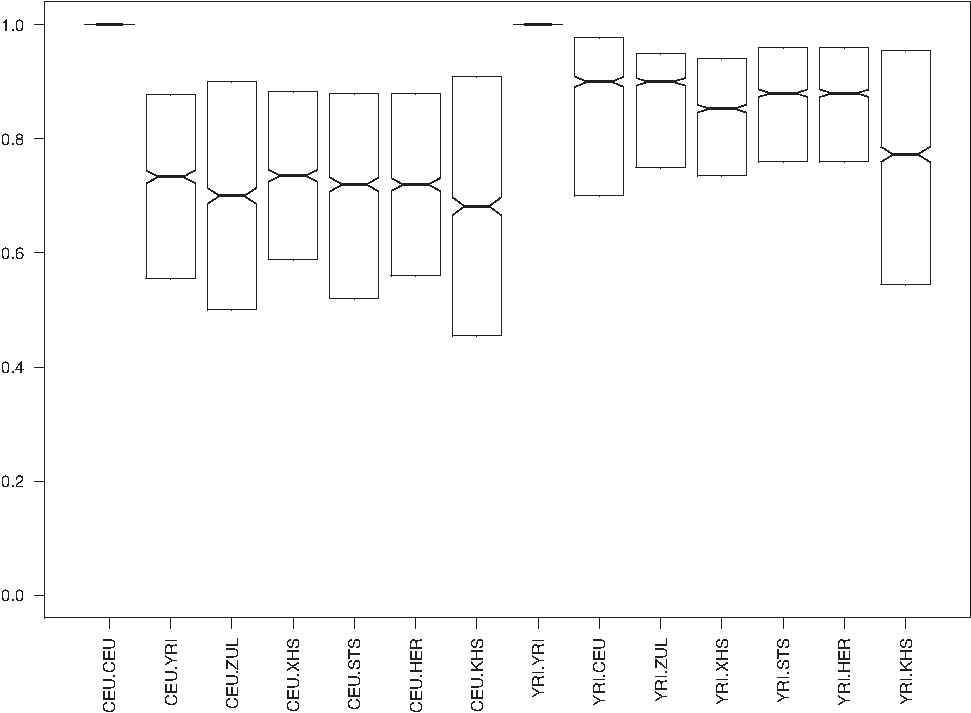

Supplement: S3 Fig — (TIFF) [file pgen.1005052.s003.tiff]

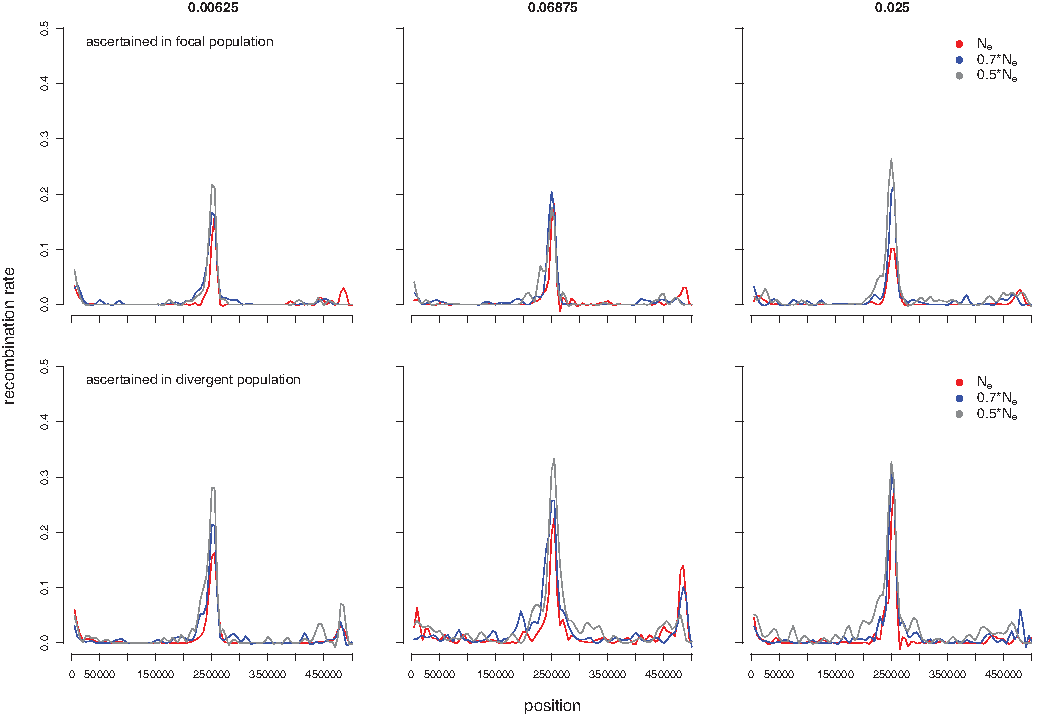

Supplement: S4 Fig — (TIFF) [file pgen.1005052.s004.tiff]

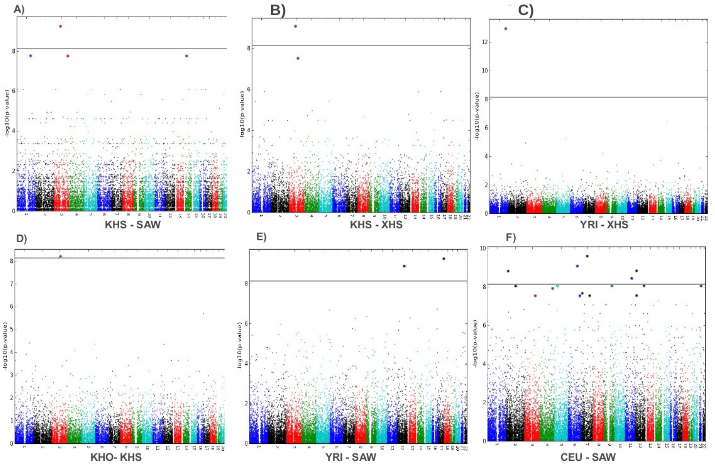

Supplement: S5 Fig — (TIFF) [file pgen.1005052.s005.tiff]

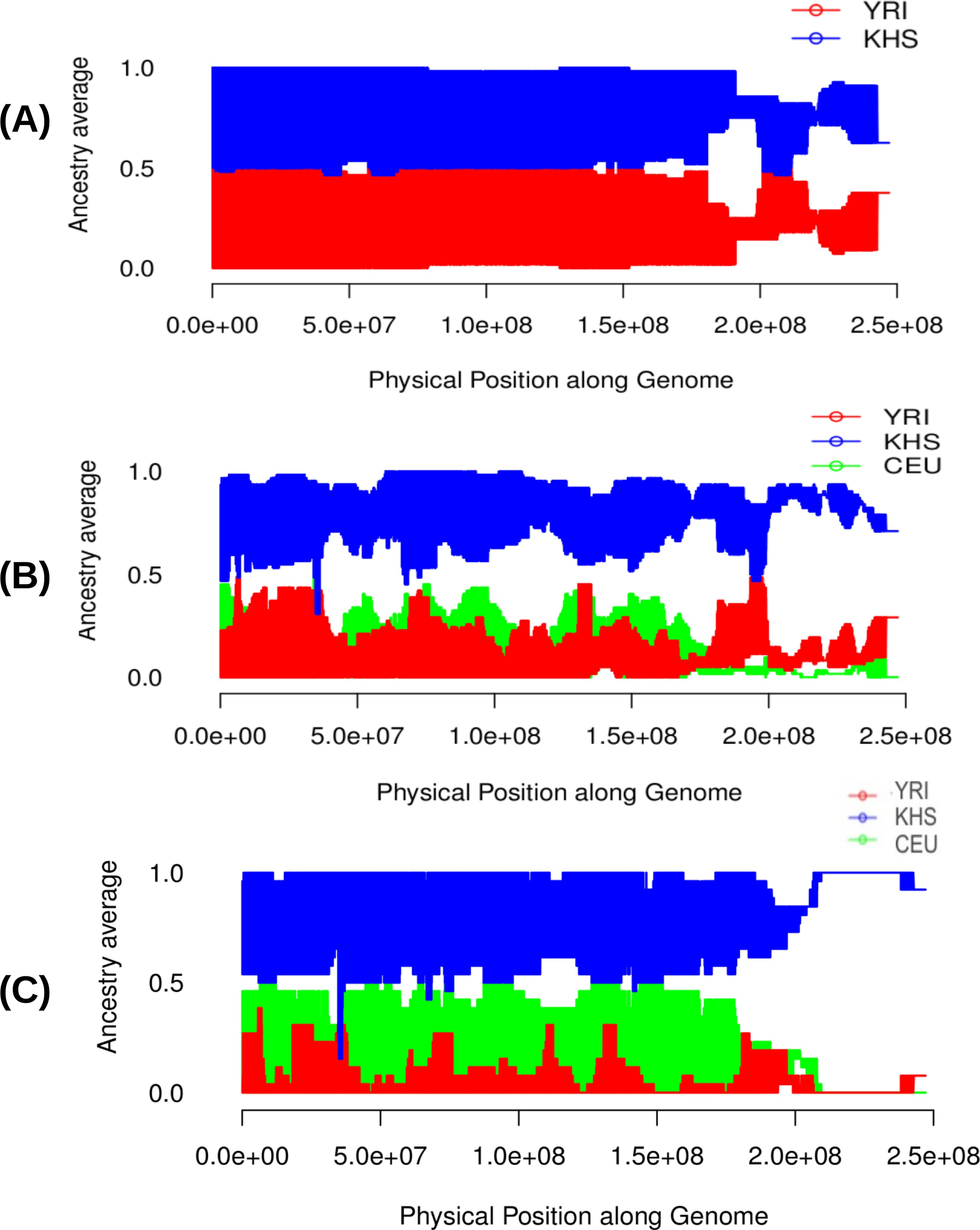

Supplement: S6 Fig — Plot (A-C) consist of 47, 864 randomly selected SNPs along the entire genome. (A) Ancestry segments in Xhosa. (B) Ancestry segments in ‡Khomani. (C) Ancestry segments in Sandawe. (TIFF) [file pgen.1005052.s006.tiff]

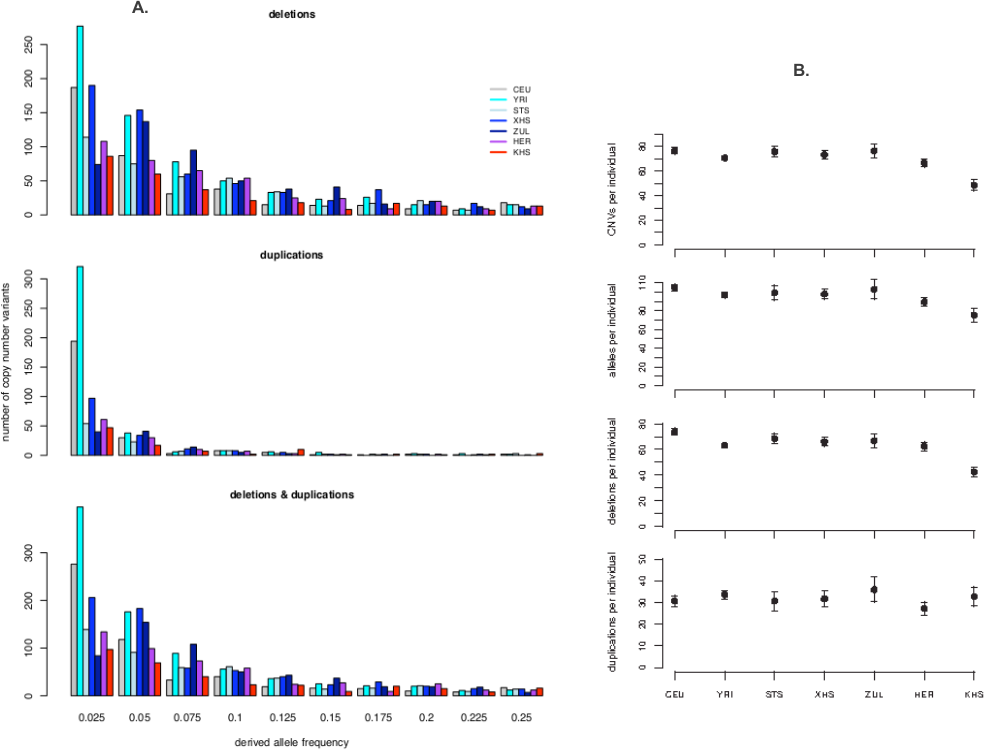

Supplement: S7 Fig — (B) The number of CNVs per individual in each of the southern African populations. (TIFF) [file pgen.1005052.s007.tiff]
