# Supplementary material for: A Genomic Portrait of Haplotype Diversity and Signatures of Selection in Indigenous Southern African Populations
Source: PLoS Genet. 2015 Mar 26;11(3):e1005052. doi: 10.1371/journal.pgen.1005052 (PMC4374865; doi:10.1371/journal.pgen.1005052)
Supplement: S1 Table — The southern Bantu-speakers in this study are represented by the Sotho-Tswana (STS) inhabiting the central plateau of southern Africa; the Nguni, represented by Zulu (ZUL), Xhosa (XHS) speakers, inhabiting KwaZulu Natal on the east coast and the Eastern Cape, and the Herero (HER) inhabiting northern Namibia, respectively (S1 Table). The eastern Bantu-speakers are mostly populations inhabiting the central lake regions and the east coast of Africa. (DOC) [file pgen.1005052.s008.doc]

| **Population** | **Description** | **n** | **Source** |
| --- | --- | --- | --- |
| **African: Bantu-Niger (Central)** | | | |
| Kaba | Kaba from Central African Republic | 17 | Brenna et al. 2011 |
| Fang | Fang from Southern Gabon and Cameroon | 15 | Brenna et al. 2011 |
| Kongo | Kongo from Democratic Republic of Congo | 9 | Brenna et al. 2011 |
| Hausa | Hausa from Niger | 12 | Brenna et al. 2011 |
| Bulala | Bulala from Malawi | 15 | Brenna et al. 2011 |
| Mada | Mada from Cameroon | 12 | Brenna et al. 2011 |
| Bamoun | Bamoun from Cameroon | 18 | Brenna et al. 2011 |
| Fulani | Fulani from Niger | 12 | Brenna et al. 2011 |
| **African: Eastern Bantu-speaking** | | | |
| MKK | Maasai in Kinyawe, Kenya | 119 | HapMap 3 |
| HAZ | Hadza from Tanzania | 17 | Brenna et al. 2011 |
| LWK | Luhya in Webuye, Kenya | 107 | HapMap 3 |
| SAW | Sandawe from Tanzania | 28 | Brenna et al. 2011 |
| BAN | Bantu-Kenya | 18 | HGDP |
| **African:Southern Bantu-speaking** | | | |
| XHS | Xhosa-South-Africa | 28 | - |
| ZUL | Zulu-South-Africa | 17 | - |
| HER | Herero , South Africa-Namibia | 22 | - |
| STS | Sotho-Tswana,South Africa | 25 | - |
| XHS2 | Xhosa from Southeastern South Africa | 11 | HGDP |
| **African: Khoesan** | | | |
| SAN | Namibia Khoisan | 5 | HGDP |
| KHS | Namibia Khoisan | 24 | - |
|  |  |  |  |
| **African: Bantu-Niger (Western)** | | | |
| Brong | Brong from mid-western Ghana | 8 | Brenna et al. 2011 |
| Igbo | Igbo from southeastern Nigeria | 15 | Brenna et al. 2011 |
| MAN | Mandenka from Senegal | 22 | HGDP |
| YOR | Yoruba in Ibadan, Nigeria | 21 | HGDP |
| YRI | Yoruba in Ibadan, Nigeria | 147 | HapMap 3**/1000G** |
| **Pygmies from Africa** | | | |
| MPG | Mbuti Pygmies from Central Africa | 13 | HGDP |
| BGP | Biaka Pygmies from central Africa | 22 | HGDP |
| **African: Afroasiatic** | | | |
| ALG | Algeria | 19 | Brenna et al. 2011 |
| MRC_N | North Morocco | 18 | Brenna et al. 2011 |
| LBY | Libya | 17 | Brenna et al. 2011 |
| MRC_S | South Morocco | 16 | Brenna et al. 2011 |
| TNS | Tunisia | 18 | Brenna et al. 2011 |
| MOZ | Mozabite | 29 | Brenna et al. 2011 |
| EGT | Egypt | 19 | Brenna et al. 2011 |
| SAH_occ | Sahara Occidental | 18 | Brenna et al. 2011 |
| **Non-African:** | | | |
| CEU | Utah residents with Northern and Western European ancestry from the CEPH collection | 112 | HapMap 3 |
| PAL | Palestinian-Israel-Central | 46 | HGDP |
| CAM | Cambodian-Cambodia, East Asia | 10 | HGDP |
| ASW | African ancestry in Southwest USA | 49 | HapMap 3 |
| CHB | Han Chinese in Beijing, China | 137 | HapMap 3/1000G |
| JPT | Japanese in Tokyo, Japan | 113 | HapMap 3/1000G |
| GIH | Gujarati Indians in Houston, Texas | 93 | HapMap 3/1000G |
