# Supplementary material for: A Genomic Portrait of Haplotype Diversity and Signatures of Selection in Indigenous Southern African Populations
Source: PLoS Genet. 2015 Mar 26;11(3):e1005052. doi: 10.1371/journal.pgen.1005052 (PMC4374865; doi:10.1371/journal.pgen.1005052)
Supplement: S3 Table — Shown are all populations with at least one negative f3 statistic, the names of the putative mixing populations (population1 and 2, not necessarily the populations that actually mixed historically) that give rise to the minimum f3 statistic, the value of the statistic, and its standard error. (DOC) [file pgen.1005052.s010.doc]

| **Population 1** | **Population 2** | **“Admixed” Population** | **f_3** | **Std.err** | **Z** |
| --- | --- | --- | --- | --- | --- |
| KHS | STS | KHO | -0.004 | 0.0003 | -13.5 |
| KHS | YRI | KHO | -0.01 | 0.0003 | -21.4 |
| KHS | CEU | KHO | -0.03 | 0.0006 | -43.3 |
| STS | CEU | KHO | 0.017 | 0.0008 | 21.9 |
| KHS | YRI | BUS | -0.012 | 0.0005 | -22.7 |
| KHS | STS | BUS | -0.009 | 0.0005 | -20.3 |
| KHS | CEU | BUS | -0.013 | 0.0008 | -16.6 |
| YRI | CEU | BUS | 0.0701 | 0.0014 | 50.7 |
| LWK | CEU | MKK | -0.016 | 0.0003 | -50.4 |
| KHS | CEU | MKK | -0.018 | 0.0006 | -27.4 |
| KHS | YRI | MKK | 0.0128 | 0.0004 | 32.5 |
| YRI | STS | KHO | 0.0446 | 0.0006 | 72.5 |
| MKK | CEU | LWK | 0.0322 | 0.0005 | 68.5 |
| KHS | CEU | LWK | 0.0016 | 0.0007 | 2.05 |
| YRI | CEU | LWK | -0.003 | 0.0003 | -11.6 |
| YRI | KHS | STS | -0.013 | 0.0003 | -43.7 |
| YRI | KHS | ZUL | -0.008 | 0.0003 | -23.7 |
| YRI | SAN | KHS | -0.008 | 0.0005 | -14.9 |
| YRI | KHS | SAN | -0.005 | 0.0008 | -6.3 |
| YRI | KHS | HER | -0.011 | 0.0005 | -20.7 |
| YRI | CEU | ZUL | 0.007 | 0.0005 | 14.9 |
| YRI | CEU | KHS | 0.109 | 0.0016 | 67.5 |
| YRI | KHS | XHS | -0.011 | 0.0003 | -36.6 |
| YRI | CEU | XHS | 0.005 | 0.0004 | 13.1 |
| KHS | CEU | XHS | -0.007 | 0.0007 | -9.6 |
