# Supplementary material for: A Genomic Portrait of Haplotype Diversity and Signatures of Selection in Indigenous Southern African Populations
Source: PLoS Genet. 2015 Mar 26;11(3):e1005052. doi: 10.1371/journal.pgen.1005052 (PMC4374865; doi:10.1371/journal.pgen.1005052)
Supplement: S4 Table — These outliers show possible obstacles to migration. (DOC) [file pgen.1005052.s011.doc]

| **Population 1** | **Population 2** | **Obstacles** |
| --- | --- | --- |
| STS | YRI | Kalahari Desert, Rainforest |
| KHS | ZUL | Kalahari Desert, Mountain |
| KHS | HER | Kalahari Desert, Mountain |
| XHS | YRI | Kalahari Desert |
| XHS | LWK | Mountain, Kalahari Desert |
| ZUL | YRI | Kalahari Desert |
| SAN | HER | Kalahari Desert, Mountain |
| ZUL | SAN | Kalahari Desert, Mountain |
| HER | SAN | Kalahari Desert, Mountain |
