# Supplementary material for: A Genomic Portrait of Haplotype Diversity and Signatures of Selection in Indigenous Southern African Populations
Source: PLoS Genet. 2015 Mar 26;11(3):e1005052. doi: 10.1371/journal.pgen.1005052 (PMC4374865; doi:10.1371/journal.pgen.1005052)
Supplement: S5 Table — (DOC) [file pgen.1005052.s012.doc]

| 1. **Panel** | **Total # blocks** | **SNPs per block** | **Mean block size (bp)** |
| --- | --- | --- | --- |
|  |  |  |  |
| CEU | 71418 | 6.19 | 29 287 |
| CHB | 62475 | 6.15 | 30 707 |
| JPT | 61947 | 6.26 | 30 776 |
| YRI | 79006 | 4.26 | 14 065 |
| XHS | 48229 | 4.20 | 19 294 |
| KHS | 27440 | 4.01 | 14 419 |
| STS | 34705 | 4.16 | 13 871 |
| ZUL | 25672 | 4.29 | 14 141 |
| HER | 37215 | 4.13 | 15 753 |
|  |  |  |  |
