# Supplementary material for: A Genomic Portrait of Haplotype Diversity and Signatures of Selection in Indigenous Southern African Populations
Source: PLoS Genet. 2015 Mar 26;11(3):e1005052. doi: 10.1371/journal.pgen.1005052 (PMC4374865; doi:10.1371/journal.pgen.1005052)
Supplement: S6 Table — Values in parentheses indicate the number of recombination hotspots shared with CEU and YRI respectively, for each of the southern African populations included in this study. (DOC) [file pgen.1005052.s013.doc]

|  | **CEU** | **YRI** | **STS** | **XHS** | **ZUL** | **HER** | **KHS** |
| --- | --- | --- | --- | --- | --- | --- | --- |
|  |  |  |  |  |  |  |  |
| **1** | 562 | 272 | 256 (69,69) | 262 (95,68) | 241 (81,56) | 301 (82,58) | 228 (40,45) |
| **2** | 592 | 327 | 259 (84,63) | 293 (90,68) | 287 (82,70) | 301 (88,71) | 241 (63,49) |
| **3** | 536 | 264 | 207 (70,59) | 261 (101,71) | 215 (67,54) | 262 (85,51) | 186 (37,31) |
| **4** | 503 | 240 | 240 (65,62) | 268 (107,71) | 234 (76,62) | 265 (75,65) | 207 (54,53) |
| **5** | 531 | 258 | 221 (79,53) | 266 (105,80) | 233 (88,60) | 287 (101,73) | 206 (62,42) |
| **6** | 506 | 255 | 218 (68,54) | 253 (102,61) | 239 (74,62) | 250 (98,63) | 220 (58,52) |
| **7** | 441 | 205 | 212 (78,62) | 231 (86,61) | 185 (62,44) | 202 (69,53) | 189 (55,44) |
| **8** | 461 | 234 | 179 (73,63) | 211 (92,71) | 163 (58,52) | 198 (84,71) | 170 (54,58) |
| **9** | 391 | 183 | 148 (62,50) | 161 (78,55) | 131 (57,43) | 165 (55,43) | 128 (47,29) |
| **10** | 451 | 220 | 190 (63,57) | 201 (87,60) | 187 (73,50) | 210 (90,57) | 150 (37,38) |
| **11** | 423 | 212 | 174 (62,65) | 213 (85,66) | 197 (64,60) | 209 (81,71) | 171 (40,35) |
| **12** | 394 | 191 | 180 (62,53) | 194 (77,61) | 150 (49,42) | 206 (66,52) | 130 (38,29) |
| **13** | 334 | 188 | 134 (65,51) | 175 (78,65) | 140 (56,46) | 151 (63,61) | 143 (47,47) |
| **14** | 325 | 135 | 103 (46,37) | 119 (65,49) | 98 (45,30) | 124 (56,38) | 112 (40,31) |
| **15** | 269 | 108 | 63 (29,24) | 86 (39,30) | 69 (37,23) | 98 (42,33) | 56 (23,12) |
| **16** | 253 | 84 | 71 (32,26) | 86 (33,29) | 62 (28,21) | 80 (33,30) | 69 (21,15) |
| **17** | 185 | 59 | 47 (17,15) | 73 (32,12) | 35 (14,6) | 80 (28,13) | 61 (18,11) |
| **18** | 303 | 117 | 83 (39,36) | 108 (50,42) | 87 (47,40) | 120 (53,43) | 86 (27,27) |
| **19** | 89 | 21 | 21 (8,5) | 35 (12,6) | 28 (10,5) | 41 (12,7) | 34 (5,3) |
| **20** | 258 | 92 | 65 (30,23) | 82 (38,28) | 64 (28,23) | 105 (51,33) | 70 (25,17) |
| **21** | 150 | 60 | 35 (15,14) | 47 (25,18) | 33 (16,17) | 39 (22,19) | 43 (16,15) |
| **22** | 101 | 20 | 15 (6,5) | 24 (12,4) | 10 (1,2) | 26 (6,4) | 24 (8,2) |
|  | **8058** | **3745** | **3121 (1122, 946)** | **3649 (1489, 1076)** | **3088 (1113, 868)** | **3720 (1340, 1009)** | **2924 (815, 685)** |
