# Supplementary material for: A Genomic Portrait of Haplotype Diversity and Signatures of Selection in Indigenous Southern African Populations
Source: PLoS Genet. 2015 Mar 26;11(3):e1005052. doi: 10.1371/journal.pgen.1005052 (PMC4374865; doi:10.1371/journal.pgen.1005052)
Supplement: S7 Table — Power is estimated as the proportion of simulated datasets in which a recombination hotspot is inferred with strength 50 times the background recombination rate, and which lies within 25kb of the simulated hotspot. (DOC) [file pgen.1005052.s014.doc]

|  |  | τ = **0.00625** | τ = **0.06875** | τ = **0.025** |
| --- | --- | --- | --- | --- |
|  |  |  |  |  |
| (a) | 0.7**N_e_* | 0.36 | 0.37 | 0.34 |
|  | 0.5**N_e_* | 0.38 | 0.26 | 0.34 |
|  | *N_e_* | 0.32 | 0.38 | 0.29 |
|  |  |  |  |  |
| (b) | 0.7**N_e_* | 0.42 | 0.30 | 0.39 |
|  | 0.5**N_e_* | 0.37 | 0.25 | 0.38 |
|  | *N_e_* | 0.39 | 0.29 | 0.42 |
|  |  |  |  |  |
