# Supplementary material for: A Genomic Portrait of Haplotype Diversity and Signatures of Selection in Indigenous Southern African Populations
Source: PLoS Genet. 2015 Mar 26;11(3):e1005052. doi: 10.1371/journal.pgen.1005052 (PMC4374865; doi:10.1371/journal.pgen.1005052)
Supplement: S8 Table — (DOC) [file pgen.1005052.s015.doc]

| **Panel** | **Number of monomorphic SNPs** | **Proportion monomorphic** |
| --- | --- | --- |
|  |  |  |
| CEU | 116 114 | 0.145 |
| YRI | 59 593 | 0.075 |
| STS | 76 357 | 0.096 |
| XHS | 32 515 | 0.041 |
| ZUL | 87 310 | 0.109 |
| HER | 67 408 | 0.084 |
| KHS | 157 804 | 0.196 |
|  |  |  |
