# Supplementary material for: A Genomic Portrait of Haplotype Diversity and Signatures of Selection in Indigenous Southern African Populations
Source: PLoS Genet. 2015 Mar 26;11(3):e1005052. doi: 10.1371/journal.pgen.1005052 (PMC4374865; doi:10.1371/journal.pgen.1005052)
Supplement: S9 Table — Values in parentheses are standard errors. (DOC) [file pgen.1005052.s016.doc]

| **Population** | ***r*** | | | ***h*** | |
| --- | --- | --- | --- | --- | --- |
| CEU | -0.0253 | (0.0007) | 0.736 | (0.0003) |  |
| YRI | -0.0350 | (0.0005) | 0.717 | (0.0002) |  |
| STS | -0.0428 | (0.0015) | 0.728 | (0.0013) |  |
| XHS | -0.0179 | (0.0016) | 0.725 | (0.0011) |  |
| ZUL | -0.0433 | (0.0022) | 0.728 | (0.0014) |  |
| HER | -0.0288 | (0.0020) | 0.727 | (0.0013) |  |
| KHS | 0.0922 | (0.0034) | 0.772 | (0.0013) |  |
