# Supplementary material for: A Genomic Portrait of Haplotype Diversity and Signatures of Selection in Indigenous Southern African Populations
Source: PLoS Genet. 2015 Mar 26;11(3):e1005052. doi: 10.1371/journal.pgen.1005052 (PMC4374865; doi:10.1371/journal.pgen.1005052)
Supplement: S1 Text — (DOC) [file pgen.1005052.s017.doc]

**Haplotype phasing, Linkage disequilibrium and Imputation**

We used fastPHASE [71] (with default parameters) to estimate chromosome-wide haplotypes for each of the 22 autosomes. The optimal number of clusters, which minimized switch error, was chosen using a cross-validation approach as previously described [68]. Inferred haplotypes were processed using Haploview [72] to estimate linkage disequilibrium (LD) statistics and haplotype block lengths [73]. We performed an imputation study using impute2 [74], in order to establish the gain in data acquired from the use 1000 Genome (http://www.1000genomes.org/data) over the previous Affymetrix Genome-Wide Human SNP Array 6.0 (approximately 0.29 SNPs/KB). Due to the strong levels of linkage disequilibrium observed between adjacent markers, missing genotypes could be imputed given knowledge of global haplotypes and the genotypes at flanking SNPs [71,74]. However, the success of imputation is likely dependent on both the levels of linkage disequilibrium in the study population, and on the divergence of the study population from which the known haplotypes are drawn. For each analysis panel we simulated missing SNPs by removing all SNPs not present on the Affymetrix 900k array, and imputed missing genotypes at these loci using the program Impute [74] with Yoruba (YRI) or European (CEU) haplotypes from 1000 Genome. Imputation success rates were determined as the percentage of correctly imputed genotypes for each of the analysis panels.

**References**

71 Browning SR and Browning BL (2007) Rapid and accurate haplotype phasing and missing data inference for whole genome association studies using localized haplotype clustering. Am. J. Hum. Genet. 81:1084-1097.

72. Barrett JC, Fry B, Maller J, Daly MJ (2005) Haploview: analysis and visualization of LD and haplotype maps. Bioinformatics 21: 263-265.

73. Gabriel SB, Schaffner SF, Nguyen H, Moore JM, Roy J, et al. (2002) The structure of haplotype blocks in the human genome. Science 5576, 2225-2229.

74. Marchini J, Howie B, Myers S, McVean G, Donnelly P (2007).

A new multipoint method for genome-wide association studies by imputation of genotypes. Nat Genet 39: 906-913.
