# Supplementary material for: A Genomic Portrait of Haplotype Diversity and Signatures of Selection in Indigenous Southern African Populations
Source: PLoS Genet. 2015 Mar 26;11(3):e1005052. doi: 10.1371/journal.pgen.1005052 (PMC4374865; doi:10.1371/journal.pgen.1005052)
Supplement: S2 Text — (DOC) [file pgen.1005052.s018.doc]

**Simulations**

We conducted a simulation study in order to further explore the potential effects of ascertainment bias on haplotype block size estimation, the decay of linkage disequilibrium and the detection of recombination hotspots. The ascertainment scheme in our analysis differs from that in HapMap [5], where the markers were both ascertained and applied in the same populations. Rather, in our study marker selection was performed in the HapMap populations and applied to independent populations of varying divergence from the ascertained populations. Since the ascertainment scheme used to select HapMap SNPs, and thus for the Affymetrix Genome-Wide Human SNP Array 6.0, is too complex to match exactly [5], we simulate a much simpler scheme. We used a coalescent model [75] to simulate 100 replicates of 50 chromosomes in each of two equally sized populations that diverged at some time,$\tau$, in the past, where $\tau$ is measured in 4N_e_ generations. We assume a generation time of 20 years and effective population size of N_e_ = 10,000, and set $\tau$ = 0.00625, 0.06875 and 0.025 corresponding to 250, 2750 and 10,000 generations before the present. These dates correspond to the timing of the expansion of Bantu-speaking populations from West Africa (circa 5000 ybp, [39,40]), and both the earliest (circa 200,000 ybp [39,40]), and latest (circa 55,000 ybp ) estimates of the splitting of southern African Khoe-San click-speaking populations from an east African ancestral population. The population scaled recombination rate, 4N_e_r, was set to 20, corresponding to 2.5x10^-8^ recombination events per site per meiosis. These coalescent population parameters were chosen so as to represent an average 500kb region with a 5kb SNP spacing [42,75]. In addition to a spatially constant recombination rate, we simulated recombination hotspots using msHOT [75]. A recombination hotspot with strength equal to 100 times the background recombination rate was simulated in a 5kb window at the centre of the 500kb region. These simulations were designed to test the effects of both ascertainment bias and population bottlenecks on recombination hotspot estimation (as in [76]). Thus we applied a population bottleneck at the time of population divergence ($\tau$ = 0.00625, 0.06875 and 0.025), of either 30% or 50% of the ancestral effective population size, to the population from which subsequent samples were drawn. Each simulation set, with and without recombination hotspots, comprised 100 independent replicates. Marker ascertainment was performed in both populations with a random discovery sample of 5 drawn from the respective population. Ascertained markers from both populations were applied to one of the populations with the purpose of identifying the effects of marker ascertainment in divergent populations. All physical positions are reported in hg19 (b37) human genome coordinates.

**References**

75. Hellenthal G, Stephens M (2007) msHOT: Modifying Hudson's ms simulator to incorporate crossover and gene conversion hotspots. Bioinformatics 23: 520-521.

76. McVean G LDHat 2.1 http://www.stats.ox.ac.uk/∼mcvean/Ldhat/.
