# Supplementary material for: A Genomic Portrait of Haplotype Diversity and Signatures of Selection in Indigenous Southern African Populations
Source: PLoS Genet. 2015 Mar 26;11(3):e1005052. doi: 10.1371/journal.pgen.1005052 (PMC4374865; doi:10.1371/journal.pgen.1005052)
Supplement: S3 Text — (DOC) [file pgen.1005052.s019.doc]

**Imputation of missing data**

To investigate imputation accuracy, we removed same number of common SNPs present on Affymetrix 900k array from each southern African population, and imputed them as missing genotypes using Yoruba (YRI) or European (CEU) haplotypes from 1000 Genome Project. Imputation was accomplished by the IMPUTE program [74], using equal haplotypes from both YRI and CEU. Imputation success rates were determined as the percentage of correctly imputed genotypes (typed genotypes (that was removed) versus imputed genotypes) for each of the analysis panels. The accuracy of imputation of southern African genotypes using European (CEU) haplotypes is significantly lower than when Yoruba haplotypes are used (Figure S3). The greatest accuracy is achieved when missing SNPs in the Herero population are imputed using known Yoruba haplotypes (85.22%), followed by the remaining Bantu-speaking populations (ZUL: 85.03%, XHS: 83.34%, STS: 83.20%). Interestingly, the imputation accuracy of European SNPs using Yoruba haplotypes (81.06%) exceeds that of Khoe-San (73.65%). This accuracy of imputation of European genotypes using African haplotypes (YRI.CEU in Figure S3) is comparable to that of southern African genotypes with African haplotypes, probably since haplotypic diversity in Europeans is a subset of African haplotypic diversity [35, 39, 40]. In general, the imputation accuracy is high, consistent with previous imputation results [77], when appropriate haplotypes are used. The Yoruba population appears to be appropriate for imputation, at least for some of the southern Bantu-speaking groups included in the study (STS, ZUL, HER and XHS), but less so for the San for whom imputation accuracy is significantly lower than for other African populations (Figure S3). Xhosa (XHS) also have lower imputation accuracy, compared with other Bantu-speaking groups, potentially the combined result of San admixture (see below), and consistently shorter regions of linkage disequilibrium across all chromosomes (Figure 3(B)).

**Reference**

74. Marchini J, Howie B, Myers S, McVean G, Donnelly P (2007). A new multipoint method for genome-wide association studies by imputation of genotypes. Nat Genet 39: 906-913.
