# Supplementary material for: A Genomic Portrait of Haplotype Diversity and Signatures of Selection in Indigenous Southern African Populations
Source: PLoS Genet. 2015 Mar 26;11(3):e1005052. doi: 10.1371/journal.pgen.1005052 (PMC4374865; doi:10.1371/journal.pgen.1005052)
Supplement: S4 Text — (DOC) [file pgen.1005052.s020.doc]

**Fine Scale recombination mapping**

We estimated fine-scale recombination rate variation [65] in all 22 autosomes for each southern African population independently, and for unrelated individuals in each of two HapMap populations (CEU & YRI) for comparative purposes. The 798 807 SNPs, shared between HapMap and our study, were used. We used *interval* from the LDHat 2.1 package [76] to estimate the recombination rate across each chromosome, for each population. Parameter settings included a total chain length of 1,000,000 samples, with a 100,000 burn-in, a recombination rate-switching penalty between successive SNPs of 5, and a population-scaled mutation rate per locus ( $\theta=\text{4N}_{e}\mu$) of 0.001. Putative recombination hotspots were identified at SNPs at which the estimated population-scaled recombination rate ($\rho=\text{4N}_{e}r$) was more than 5 times greater than the average chromosome-wide recombination rate. We utilized this empirical outlier approach rather than coalescent simulations [76], since the latter are problematic, to parametrize given the complex demographic histories of the populations included in this study.

**Results**

We assess the impact of fine-scale recombination events to differences in linkage disequilibrium patterns using a coalescent-based method [75]. The southern African Bantu-speaking populations share proportionally more recombination hotspots with both Yoruba (YRI) and European (CEU) than with the Ju\’hoansi (KHS) (Figure 4, supplementary Table S6), where a shared hotspot is identified as a region with greater than five times the background recombination rate within a 10kb window. The proportion of hotspots shared between southern Africans and both European (CEU) and Yoruba (YRI) samples was generally low (Figure 4). This may be the result of larger sample sizes in these populations, compared to the southern African data, and thus less power to detect recombination hotspots in the southern African populations. However, the absolute number of recombination hotspots identified in the Yoruba (YRI) is similar to that in the southern African populations (S6 Table), suggesting that the low levels of hotspot sharing may not be a sample size effect. Although the coalescent method has been shown to be robust to deviations from a neutral coalescent model [75], greater power to detect recombination rate variation is achieved with populations that have experienced bottlenecks; probably since the increase in background linkage disequilibrium makes hotspots prominent [75]. Our simulations (S2 Text) confirm this, and further show that the power to detect a recombination hotspot is dependent on the severity of the population bottleneck (S4 Fig, top panel; S7 Table (A)). This is particularly apparent when hotspots are old (i.e. τ = 0.025), potentially since non-bottlenecked populations would have proportionally more diversity than bottlenecked populations in old versus recent bottlenecks. Proportionally more recombination occurs at hotspots in Caucasians (CEU) compared to African populations (S4 Fig). These results are consistent with the relatively greater proportion of hotspots shared between southern African populations and Caucasians (CEU), than between the former and Yoruba (YRI) (Figure 4). Recombination hotspot simulations (S2 Text ) demonstrated increased power to detect recombination hotspots when markers were ascertained in divergent populations (S4 Fig, bottom panel), compared to markers ascertained in the genotyped population. This increased power is particularly apparent when the simulated bottleneck is old (S4 Fig, bottom panel; S7 Table (B). Whilst these results are of interest it is not clear why such an ascertainment scheme would improve the power to detect recombination hotspots. To test whether increased frequency of low frequency and monomorphic SNPs improves the power to detect recombination hotspots, we removed SNPs with minor allele frequencies of less than 0.05, 0.10 and 0.15 and repeated fine scale recombination mapping. The result was the same irrespective of the minor allele frequency limit (S2 Fig(B)). In general, our empirical analyses indicate that few recombination hotspots are shared between southern Africans and the HapMap populations, with San being the most extreme. However, the extent to which these results are due to real differences in the fine-scale pattern of recombination between populations, or the result of marker ascertainment is uncertain (see method in the section above).

References

75. Hellenthal G, Stephens M (2007) msHOT: Modifying Hudson's ms simulator to incorporate crossover and gene conversion hotspots. Bioinformatics 23: 520-521.

76. McVean G LDHat 2.1 http://www.stats.ox.ac.uk/∼mcvean/Ldhat/.
