# Supplementary material for: A Genomic Portrait of Haplotype Diversity and Signatures of Selection in Indigenous Southern African Populations
Source: PLoS Genet. 2015 Mar 26;11(3):e1005052. doi: 10.1371/journal.pgen.1005052 (PMC4374865; doi:10.1371/journal.pgen.1005052)
Supplement: S5 Text — (DOC) [file pgen.1005052.s021.doc]

**Genotype and copy number calling**

Quality of hybridization intensity (CEL) files was assessed with the Dynamic Model (DM) algorithm, and only individuals for which the call rate was greater than 90% (QC > 90) were included in downstream genotype calling. Nine CEL files were removed, thus reducing sample sizes to 25 Sotho-Tswana (STS), 25 Herero (HER), 22 Ju\’hoansi San (KHS), 20 Zulu (ZUL) and 35 Xhosa (XHS). Mean CEL file QC call rates were all > 97.5% for the remaining individuals (Table S7). Probe specific intensities for each sample were normalized and summarized using the Affymetrix quantile normalization protocol. Genotypes were called using the Birdseed algorithm distributed with Affymetrix Power Tools ([http://www.affymetrix.com](http://www.affymetrix.com/estore/partners_programs/programs)). HapMap3 genotypes (CEU, GIH, ASW, CHB+JPT, YRI) were downloaded from the HapMap website ([http://www.hapmap.org](http://www.hapmap.org/)). Additional publicly available African genotypes, generated on Illumina 650Y arrays and on the Illumina Beadchip 550K, were downloaded from the Human Genome Diversity Project (HGDP; <http://hagsc.org/hgdp/files.html>) and <http://wwwevo.stanford.edu/>[repository/paper0002/](http://wwwevo.stanford.edu/repository/paper0002/) data used in [5, 77]. We used these populations for population structure analysis. Since only 50K SNPs are shared between HapMap, HGDP and the data generated in this study, we only utilized this additional public data for population structure analyses and not for other analyses that require denser SNP coverage.

Copy number was called using the Canary and Birdseye algorithms [57, 58], which call both known copy number polymorphisms (CNPs) and scan for novel copy number variants (CNVs). Briefly, the method integrates the calling of copy number variation and genotypes using both known copy number polymorphisms (Canary), and rare copy number variants (Birdseye) identified with a Hidden Markov Model (HMM) in which hidden states are copy number and observed states, hybridization intensities [57]. Default parameters, as specified in [39], were used with the exception that we did not limit the copy number models tested to those defined by the prior for the Canary algorithm. The prior of the Canary algorithm can be configured to limit the possible copy number states to those observed in HapMap3 samples. Since our populations may have novel copy number states, we configured the Canary algorithm to consider all possible states. In order to test the reliability of calling CNPs and CNVs on smaller datasets we compared results from copy number called for three of the south-eastern Bantu-speaking populations (STS, XHS, ZUL) combined (n = 80), and independently for the population with smallest sample size after quality-filtering (n = 20; ZUL). Quality filtering was performed on both the CNP (Canary) and CNV (Birdseye) calls. In the former, known copy number polymorphisms with confidence scores less than 0.1 were removed from further analysis (3-8% of CNP calls, S8 Table), whereas for CNVs individuals with sample copy number variances > 2, indicative of either noisy data or substantial copy number variation were excluded from further analysis. Quality filtering of Birdseye CNV calls removed a total of 15 individuals from subsequent copy number analysis (CEU = 1, YRI = 3, STS+XHS+ZUL = 5, HER = 2, KHS = 4). Confidence in HapMap panel CNP calls was greater than in southern African populations, even when matched for sample size, probably the result of the inclusion of trios in the former. Since the Hidden Markov Model used to detect copy number variants does not preclude distinct boundaries of copy number changes across individuals, we defined copy number variants as overlapping segments smaller than 1MB in size, with LOD scores > 5, and for which at least 10 probes were used to call the copy number state. Copy number variant loci were specified as homozygous deletion (0), heterozygous deletion (1), normal copy number (2), heterozygous duplication (3) and homozygous duplication (4). All copy number calls used in downstream analysis for the STS, XHS and ZUL panels were derived from the combined calling of these populations, in order to maximize sample size for the calling of copy number in these populations. The pooling of these populations is justified by the lack of evident population structure. CEL files generated from the HapMap populations were obtained from Affymetrix and were processed for copy number calling in the same manner for comparative purposes.

Reference

77. Cann HM, de Toma C, Cazes L, Legrand MF, Morel V, et al. (2002) A Human Genome Diversity Cell Line Panel. Science 296: 261b-262.5.
