# Supplementary material for: A Genomic Portrait of Haplotype Diversity and Signatures of Selection in Indigenous Southern African Populations
Source: PLoS Genet. 2015 Mar 26;11(3):e1005052. doi: 10.1371/journal.pgen.1005052 (PMC4374865; doi:10.1371/journal.pgen.1005052)
Supplement: S6 Text — (DOC) [file pgen.1005052.s022.doc]

**Data Filtering**

Pair-wise analysis of relatedness between individuals identified one parent-offspring or full-sibling relationship (r = 0.49) within the Khoe-San group, and was excluded; 13 possible higher order relationships (r ≈ 0.25) were also detected. These relationships result in increased mean relatedness within the Khoe-San, compared to the other populations (Table 1), however we retained them in subsequent analyses since the statistic used is less predictive of higher-order than parent-offspring and full-sibling relationships [67,68]. All other southern African populations had mean relatedness equivalent to HapMap populations in which known parent-offspring pairs were excluded (Table 1).
