# Supplementary material for: A Genomic Portrait of Haplotype Diversity and Signatures of Selection in Indigenous Southern African Populations
Source: PLoS Genet. 2015 Mar 26;11(3):e1005052. doi: 10.1371/journal.pgen.1005052 (PMC4374865; doi:10.1371/journal.pgen.1005052)
Supplement: Supplementary file 16 [file pgen.1005052.s009.doc]

	SAN	HER	STS	XHS	ZUL	YRI	MKK	LWK	HAZ	SAW	CHB+JPT	CEU
KHS	0.008±0.0010	0.069±0.0010	0.067±0.0007	0.068±0.0008	0.077±0.0009	0.101±0.0009	0.099±0.0010	0.094±0.0009	0.102±0.0015	0.089±0.0010	0.246±0.0019	0.000±0.0000
SAN		0.070±0.0017	0.068±0.0015	0.070±0.0015	0.079±0.0016	0.103±0.0016	0.102±0.0017	0.096±0.0016	0.104±0.0022	0.091±0.0017	0.249±0.0024	0.000±0.0000
HER			0.007±0.0004	0.008±0.0005	0.008±0.0005	0.012±0.0004	0.020±0.0005	0.010±0.0004	0.027±0.0011	0.019±0.0006	0.165±0.0016	0.000±0.0000
STS				0.003±0.0002	0.001±0.0002	0.013±0.0002	0.026±0.0004	0.011±0.0002	0.030±0.0010	0.024±0.0005	0.181±0.0016	0.000±0.0000
XHS					0.002±0.0002	0.014±0.0002	0.025±0.0003	0.012±0.0002	0.030±0.0010	0.023±0.0005	0.176±0.0016	0.000±0.0000
ZUL						0.010±0.0003	0.025±0.0004	0.009±0.0002	0.030±0.0010	0.024±0.0005	0.180±0.0017	0.000±0.0000
YRI							0.024±0.0003	0.007±0.0001	0.031±0.0010	0.025±0.0004	0.178±0.0016	0.000±0.0000
MKK								0.014±0.0002	0.025±0.0009	0.010±0.0003	0.138±0.0013	0.000±0.0000
LWK									0.023±0.0009	0.017±0.0004	0.168±0.0015	0.000±0.0000
HAZ										0.025±0.0010	0.175±0.0020	0.000±0.0000
SAW											0.145±0.0015	0.000±0.0000
CHB+JPT												0.000±0.0000
CEU												
