# Supplementary material for: Correction: A Genomic Portrait of Haplotype Diversity and Signatures of Selection in Indigenous Southern African Populations
Source: PLoS Genet. 2015 Jul 17;11(7):e1005363. doi: 10.1371/journal.pgen.1005363 (PMC4506067; doi:10.1371/journal.pgen.1005363)
Supplement: S1 File — (PDF) [file pgen.1005363.s001.pdf]

RESEARCH ARTICLE

A Genomic Portrait of Haplotype Diversity and Signatures of Selection in Indigenous Southern African Populations

Emile R. Chimusa[1,2], Ayton Meintjies[1], Milaine Tchanga[1], Nicola Mulder[1], Cathal Seioghe[3], Himla Soodyall[4], Rajkumar Ramesar[5]*

1 Computational Biology Group, Institute of Infectious Disease and Molecular Medicine, University of Cape Town, Cape Town, South Africa, **2** Centre for Proteomic and Genomic Research, Cape Town, South Africa, **3** School of Mathematics, Statistics and Applied Mathematics, National University of Ireland Galway, Galway, Ireland, **4** Division of Human Genetics, School of Pathology, Faculty of Health Sciences, University of Witwatersrand and the National Health Laboratory Service, Johannesburg, South Africa, **5** MRC Human Genetics Research Unit, Division of Human Genetics, Institute of Infectious Disease and Molecular Medicine, University of Cape Town, Cape Town, South Africa

* raj.ramesar@uct.ac.za

CrossMark
click for updates

Abstract

We report a study of genome-wide, dense SNP ($\sim$900K) and copy number polymorphism data of indigenous southern Africans. We demonstrate the genetic contribution to southern and eastern African populations, which involved admixture between indigenous San, Niger-Congo-speaking and populations of Eurasian ancestry. This finding illustrates the need to account for stratification in genome-wide association studies, and that admixture mapping would likely be a successful approach in these populations. We developed a strategy to detect the signature of selection prior to and following putative admixture events. Several genomic regions show an unusual excess of Niger-Kordofanian, and unusual deficiency of both San and Eurasian ancestry, which were considered the footprints of selection after population admixture. Several SNPs with strong allele frequency differences were observed predominantly between the admixed indigenous southern African populations, and their ancestral Eurasian populations. Interestingly, many candidate genes, which were identified within the genomic regions showing signals for selection, were associated with southern African-specific high-risk, mostly communicable diseases, such as malaria, influenza, tuberculosis, and human immunodeficiency virus/AIDs. This observation suggests a potentially important role that these genes might have played in adapting to the environment. Additionally, our analyses of haplotype structure, linkage disequilibrium, recombination, copy number variation and genome-wide admixture highlight, and support the unique position of San relative to both African and non-African populations. This study contributes to a better understanding of population ancestry and selection in south-eastern African populations; and the data and results obtained will support research into the genetic contributions to infectious as well as non-communicable diseases in the region.

OPEN ACCESS

Citation: Chimusa ER, Meintjies A, Tchanga M, Mulder N, Seioghe C, Soodyall H, et al. (2015) A Genomic Portrait of Haplotype Diversity and Signatures of Selection in Indigenous Southern African Populations. PLoS Genet 11(3): e1005052. doi:10.1371/journal.pgen.1005052

Received: January 28, 2014

Accepted: February 2, 2015

Published: March 26, 2015

Copyright: © 2015 Chimusa et al. This is an open access article distributed under the terms of the Creative Commons Attribution License, which permits unrestricted use, distribution, and reproduction in any medium, provided the original author and source are credited.

Funding: The funding for this project was provided by the Medical Research Council of South Africa. Research Material and Processing of the SNP6 array was done by Affymetrix, USA. The funders had no role in study design, data collection and analysis, decision to publish, or preparation of the manuscript.

Competing Interests: The authors have declared that no competing interests exist.

Author Summary

Genome-wide analysis of human populations is useful in shedding light on the evolutionary history of the human genome, with a wide range of applications from reconstructing past associations between different population histories to disease mapping. In this manuscript we report on the application of genome-wide data to southern African populations and the identification of genome-wide signatures of selection pre- and post-admixture. Several signals of selection, before and after admixture, were identified, some of which involved loci associated with human diseases, including malaria, influenza, tuberculosis and HIV/AIDS. These results may reflect adaptations of southern African populations to infectious diseases. Consistent with previous studies, this study highlights the significance of the San in the genetics of human populations, as they are distinct from the other populations in many respects i.e. haplotype structure, locations of recombination hotspots, copy number and population structure. Furthermore, our study demonstrates the admixture of the San, Bantu-speaking populations and populations of Eurasian ancestry in some of the southern and eastern African populations. It illustrates the value in correcting for this stratification in future genome-wide association studies, and suggests that a future admixture mapping in these populations would likely be warranted and successful.

Introduction

The analysis of high-throughput genotype data has revealed global patterns of human haplotype variation, casting light on the pre-history of human populations [1, 2, 3, 4, 5]. The International HapMap consortium [1,5]) and Human Genome Diversity Project (HGDP) [6], among others, have facilitated the analysis of human genome-wide variation, and linkage disequilibrium in disease association studies [1, 4, 5] and also helped refine estimates of recombination rates [7]. Comparative genome-wide genotype data among humans, Neanderthals and Chimpanzees have also shown that selection has played a significant role in human adaptation to the environment [8, 9, 10, 11]. These data have provided additional support for the African origin of modern humans [12,13] and highlight the effects of migration both within Africa and out of Africa. In general, African populations exhibit less linkage disequilibrium between adjacent markers than their non-African counterparts, consistent with a migratory bottleneck in the latter [1, 2, 5]. Such differences in the extent of linkage disequilibrium have a profound effect on the power of case-control association studies, since these studies depend largely on linkage disequilibrium between disease variants and genotyped single nucleotide polymorphisms (SNPs). Substantially more SNPs are required to capture genomic variation in African populations than populations of European ancestry [1, 5]. In addition, African populations are characterized by higher levels of genetic diversity [13, 14, 15, 16] and considerable population substructure [17, 18, 19], probably the combined result of several migration events, effective population size changes, population differentiation through genetic drift and local selective forces operating in ecologically diverse environments [18].

Hypotheses of migration within Africa based on mitochondrial DNA (mtDNA) suggest that at least three major migration events are plausible that could account for the patterns of mtDNA variation within Africa [17]; (1) the divergence of southern African San and east African populations who share the ancestral mtDNA haplogroup (L0d) and associated lineages in their maternal gene pool from an ancestral parental population circa 200 kya, (2) the establishment of west African maternal haplogroups (L1’5 & L0abf) from an east African source (circa 100 kya), and (3) the Bantu expansion from the Niger-Congo region into central, eastern and

southern Africa (< 5 kya). Although a southern African versus east African origin of modern humans cannot be fully evaluated with current data, multiple lines of evidence from mtDNA [16], Y chromosomes [20], *Alu* insertions [21], and autosomal SNPs [3] place the divergence of the San at the root of modern humans with at least 100 ky of isolation from other non-San African populations [17, 22], and relatively recent (< 5 kya) admixture with Bantu-speaking populations [16, 23, 24, 25, 26, 27], followed by subsequent admixture (< 5 kya) in the region [16, 28, 29, 30]. Given this relative isolation of present-day San in southern Africa, it is expected that many SNPs ascertained in HapMap populations may not necessarily be polymorphic in San, unless the polymorphisms arose well before the divergence of these populations. Southern Africa was occupied exclusively by the San prior to the arrival of Bantu-speaking populations within the past 1,500 years, a consequence of the Bantu-expansion out of west Africa some 5000 years ago [16, 23, 24, 25, 26, 27, 31]. Migrations across equatorial central Africa to the region of the Great Lakes in east Africa, followed by southern African migrations [16, 25] established the eastern and southeastern Bantu-speaking groups, respectively. Migrations along the west coast of Africa contributed to western and southwestern Bantu-speaking groups, the latter, currently extending to Namibia [16, 25, 26, 27, 28, 29]. According to our findings, the label "Khoe-San" represent populations resulting from the mixture of predominately San, Eurasian and Bantu-speaking populations. Over hundreds of years, indigenous San and Khoe-San communities have undergone a sharp decline in population size, largely due to warfare and diseases such as smallpox which arrived with colonialists [29, 32]. It is estimated that the population decline (i.e. 90 percent) of both San and Khoe-San populations was due to smallpox [31, 32]. Recently, Lachance et al. [33] used the whole-genome sequences of five individuals in each of three different hunter-gatherer populations, including Pygmies from Cameroon, Khoe-San-speaking Hadza and Sandawe from Tanzania, and identified several genomic regions with evidence of archaic introgression in the hunter-gatherers. In addition, Lachance et al. [33] demonstrated that distribution of the time to the most recent common ancestors for these regions was similar to that observed for introgressed regions in Europeans [33]. Ancient and relatively recent contact between immigrants from Europe, Asia and Indonesia with sub-Saharan Africans [24, 26, 34] have resulted in varying degrees of admixture between these populations. Furthermore, a recent study by Gurdasani et al. [35] presented a broad survey of polymorphisms in a novel array genotyping data set of ∼1,481 individuals from 18 self-identified ethnic/linguistic and low coverage whole genome sequencing data set of 320 individuals from 7 self-identified ethnic/linguistic in Sub-Saharan Africa, and suggested that Eurasian back migrations to Africa and contributions to ancestry has a substantial impact on differentiation among some sub-Saharan African populations. These mixtures have also contributed to shaping the gene pool of the derived populations in south-eastern Africa [28, 35]. Other disciplines, such as archaeology, history and anthropology, have given us clues about the prehistory of African populations. The study by Pickrell et al. [16] convincingly demonstrated waves of two-way admixture between Niger-Congo-speaking African and west Eurasian (European or Middle Eastern) populations to form eastern and southern African (admixed) populations. However, the role of native indigenous San in the south-eastern African region and the genetic contribution of this population to the southern and eastern African admixed populations has not been elucidated. The present study makes use of genetic markers to investigate which factors, and to what extent, they have contributed in shaping the gene pools of extant southern and eastern African populations. More specifically, we used the Affymetrix Genome-Wide Human SNP Array 6.0, to examine ∼900K SNPs and copy number variants in five indigenous populations comprising 25 Ju\'hoansi San from Namibia (KHS), southeastern Bantu-speakers [25 Sotho-Tswana (STS), 36 Xhosa (XHS), 25 Zulu (ZUL)] as well as 25 Herero (HER), a southwestern Bantu-speaking group from Namibia. These data were used in conjunction with other published data to

examine the genetic origins of southern African populations. Importantly, our study demonstrates the admixture of the indigenous San, Niger-Congo-speaking populations and populations of Eurasian ancestry in southern and eastern African populations. We have also developed two complementary approaches to identify signatures of selection prior to and following putative admixture events in the southern African populations.

Results

Sampling and Genotyping

The sample consisted of unrelated individuals belonging to the following five self-identified ethnic/linguistic populations of southern Africa: southeastern Bantu-speaking [25 Sotho-Tswana (STS), 25 Zulu (ZUL) and 36 Xhosa (XHS)], southwestern Bantu-speaking [25 Herero (HER)], and 25 Ju\'hoansi San (KHS). The Sotho-Tswana and Zulu samples were collected in Johannesburg, the Xhosa from Khayelitsha in Cape Town, the Herero from Windhoek, and the Ju\'hoansi from Tsumkwe [36]. The Blood samples were collected with the subject's informed consent, and the use of DNA samples for population genetics research was approved by both the University of the Witwatersrand and University of Cape Town. DNA samples were shipped to Affymetrix (http://www.affymetrix.com) for genotyping using the Affymetrix Genome-Wide Human SNP Array 6.0, containing 906,600 SNPs and more than 946,000 probes for the detection of copy number variation. These data were used to examine patterns of migrations, genetic ancestry and effects of selection in this study. Other populations included in this study are listed in S1 Table.

Admixture Analysis

The separation of Africans from non-Africans is clearly evident (Fig. 1 (A)); this has also been previously reported with both microsatellite data [37, 38] as well as with other SNP data [2, 3, 5]. From pairwise population genetic distance estimates, we find that there is little genetic difference among Bantu-speaking populations (S2 Table). In addition, Fig. 1 (A) shows a distinct separation of San populations (San (SAN) and Ju\'hoansi (KHS) and Khoe-San populations (Bushmen (BUS), ‡Khomani (KHO)), consistent with previous studies [16, 26, 33, 39, 40]. This result suggests Khoe-San, and both eastern and southern Bantu-speaking populations have undergone admixture. Furthermore, this result is consistent with the 3-population test [39, 40] result displayed in S3 Table, which shows clear evidence of admixture between Yoruba (YRI) and KHS in the southern Bantu (ZUL, STS, XHS). Furthermore, the ‡Khomani (KHO), and eastern Bantu-speaking populations also reflect a three-way admixture of Caucasian (CEU), Yoruba (YRI) and KHS. The results in Fig. 1 (A and D) suggest that the genetic make-up of the southeastern Bantu-speaking groups (ZUL, STS, XHS) includes ancestral contributions from Niger-Congo (26% ± 0.3%) and San populations (74% ± 0.4%). However, consistent with previous findings [40], the data in Fig. 1(B-C), suggests Niger-Congo ancestry (17% ± 1.2% and 57% ± 1.6%), San ancestry (70 ± 1.3% and 15% ± 0.4%), and notably Eurasian-related ancestry (13% ± 1% and 28% ± 2%) in the genetic make-up of ‡Khomani (KHO) and Sandawe (SAW), respectively. The admixture observed in the Khoe-San (KHO), and in the eastern African populations, (particularly) Sandawe (SAW) reflects the gene flow from Bantu-speaking agriculturalists and/or eastern African pastoralists within the past 1,200 years and sea-borne immigrants from Europe, Asia and Indonesia [33, 35, 39, 40, 41]. Our observation of Eurasian ancestry in both eastern (SAW) and southern (KHO) African populations is consistent with archaeological, genetic, climatological and linguistic data [24, 25, 26, 27, 28, 35]. Furthermore, Pickrell et al. [16] previously demonstrated multiple waves of population mixture in the history of many eastern and southern African populations, and that genetic material from Eurasians

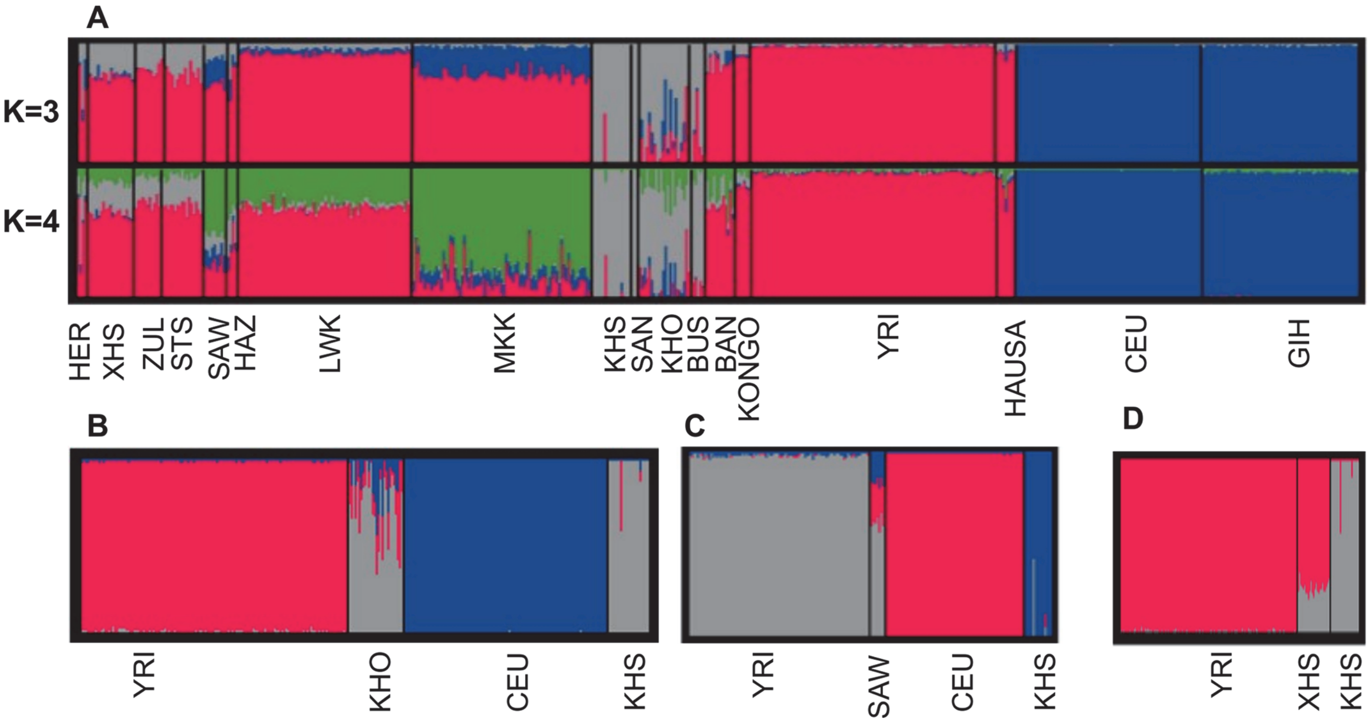

Fig 1. (A) Admixture analysis in southern African and other sub-Saharan African populations compared with Europeans and Asians. From Fig. 1A, ‡Khomani (KHO), Xhosa (XHS) and Sandawe (SAW) are 3-way, 2-way and 3-way admixed, respectively consistent with previous results [16, 33, 36]. (B-D) Admixture analyses using best proxy ancestral populations of each admixed southern African population. (B) For ‡Khomani (KHO) (C) for Sandawe (SAW) and (D) for Xhosa (XHS).

doi:10.1371/journal.pgen.1005052.g001

or related populations entered eastern Africa 2,700–3,300 years ago, and southern Africa 900–1,800 years ago [16, 41]. In addition, our study demonstrates the genetic contribution of the San population to the waves of admixture in the ancestry of the southern and eastern African populations.

Relationship between Genetic and Geographic Distance

Using the Mantel test with N = 10000 permutations (Materials and Methods), we found a significant positive correlation between genetic and geographic distance in the southern African populations (Pearson's r = 0.64; p-value = 1.0×10^{-4}; Fig. 2). To analyse more closely the outlier points in Fig. 2, we calculated the perpendicular distance between each point and the regression line. Analysing the concentration of points around the linear regression, we therefore defined outliers as points which are greater than 0.05 distance units from the regression line. When analysing the scatter plot (Fig. 2), there are 10 outlier points, which suggest possible obstacles to migration (S4 Table), assuming that populations have used the shortest path during their migrations. To assess patterns of migrations and to capture the genetic drift in southern African populations, we used a maximum likelihood tree and Gaussian approximation to the genetic drift model; implemented in Treemix [40]. We observed not only a major split between the African and European continent exhibited on this population tree, but also sub-lineages within African, and particularly within the southern African populations (S1 Fig.) which is consistent with previous results [16, 26, 34, 39, 40]. S1 Fig. (B) shows the inferred graph with three migration events, explaining the model for the relationship of southern and eastern Africans and non-Africans. This provides evidence for a shared origin for San-and Eurasian- and

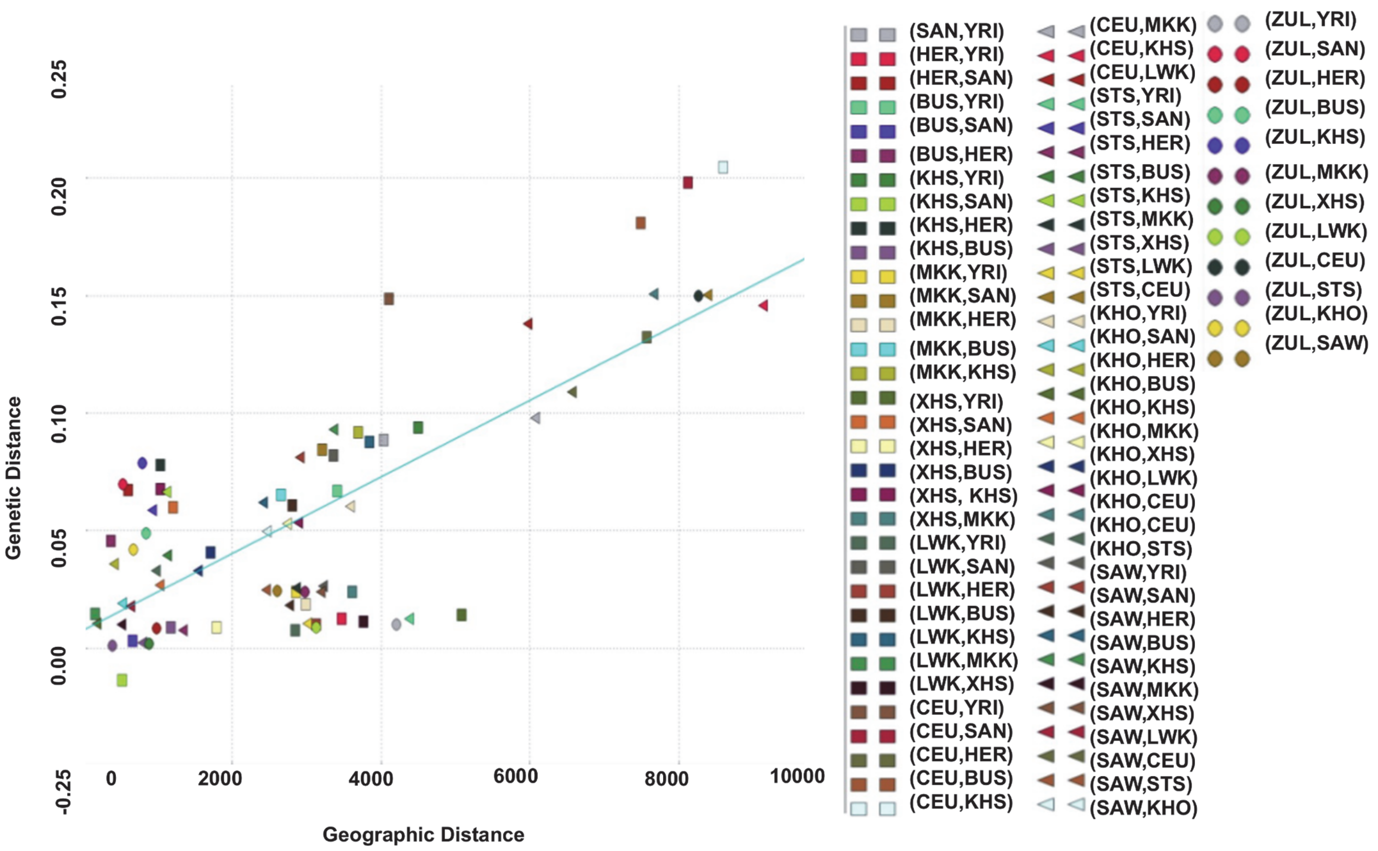

Fig 2. Relationship between genetic distances from southern African populations and their corresponding geographic distances. We identify 10 outlier points (points situated at 0.05 units from the regression line), suggesting possible obstacles to migration (see S4 Table).

doi:10.1371/journal.pgen.1005052.g002

Bantu-related populations in Sandawe (SAW) and ‡Khomani (KHO). The latter possibility would be consistent with known south-east African admixture in the Sandawe (SAW) and ‡Khomani (KHO). We clearly see four population branches in southern Africa: (i) one formed from the southern Bantu-speaking populations, which are very distinct from the Niger-Congo and eastern Bantu-speaking populations, (ii) the second group formed with eastern Bantu-speaking populations, and (iii) the third, and (iv) the fourth group formed with San (KHS+SAN) and Khoe-San (BUS+KHO), both hunter-gatherers which are quite distinct, and are split into two distinct groups, including San populations (SAN and Ju\'hoansi (KHS)) and Khoe-San populations (BUS and KHO). This is also consistent with the admixture results shown in Fig. 1, reaffirming the concordance between genetic data with geographic origins of populations and their linguistic affinities.

Haplotypes, Fine-Scale Recombination Rates and Imputation Accuracy

Consistent with previous observations [13], the mean haplotype block lengths are substantially shorter in African populations than in non-Africans (Fig. 3 (A) and S5 Table). Mean block lengths are remarkably consistent across the southern African populations in this study and easily distinguishable from the non-African block lengths. Similarly, decay of linkage disequilibrium with physical distance along the genome is rapid in southern Africans when compared with non-Africans (Fig. 3 (B)). Ascertainment biases have been shown to result in faster decay of linkage disequilibrium compared to a sample of non-ascertained markers [42]. We

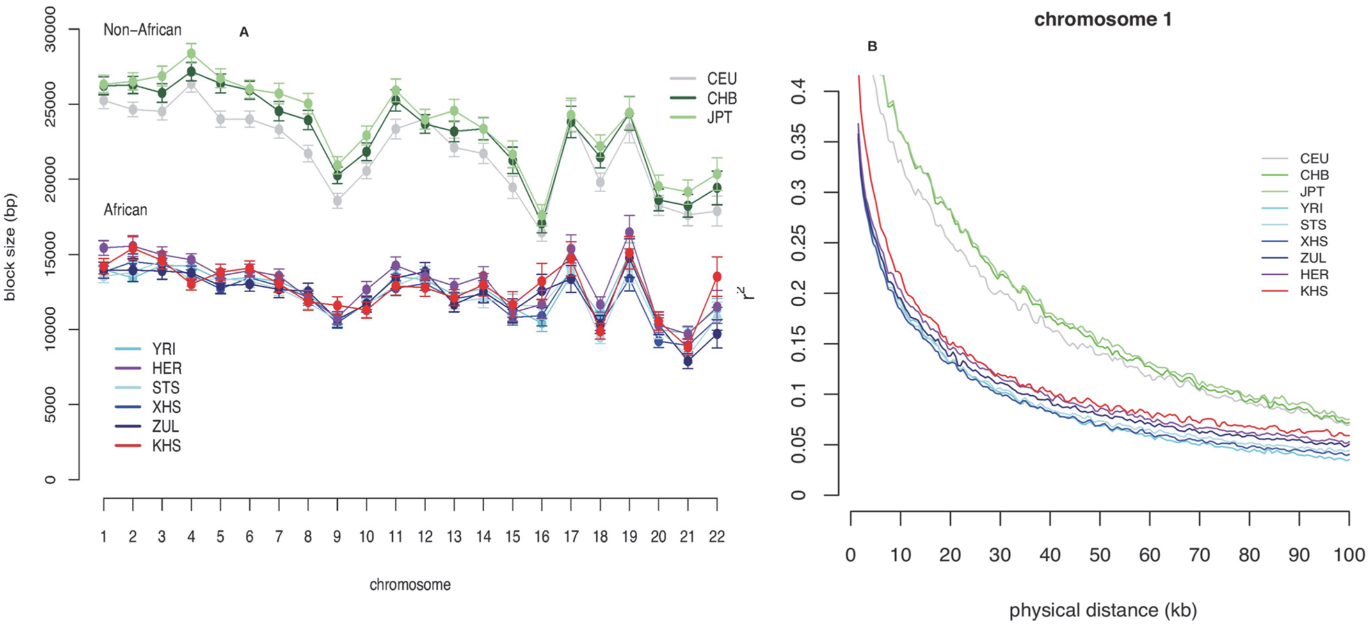

Fig 3. (A) Mean block sizes per chromosome and analysis panel. Error bars are twice the standard error of the mean. (B) Decay of linkage disequilibrium with physical distance along chromosome 1 for each analysis panel.

doi:10.1371/journal.pgen.1005052.g003

performed coalescent simulations (S1 Text and S2 Text) in order to investigate the effects of ascertainment bias when markers are ascertained in a population divergent from that in which they are genotyped. Consistent with previous reports [42], we found the rate of decay of linkage disequilibrium to be greater with ascertained SNPs (S2 Fig. (A)). Similarly, haplotype block lengths are similar, irrespective of whether markers were ascertained in the genotyped population, or in a divergent population (S2 Fig. (A)). Frequency spectra, however, differ when SNPs are ascertained in a divergent population (S2 Fig. (A)). Indeed more monomorphic SNPs, and thus lower overall SNP diversity, are evident when markers are ascertained in a population divergent from that in which they are genotyped. This is further evident in distributions of minor allele frequencies from empirical data, in which the distribution of minor allele frequencies of San more closely resembles the theoretical expectation for a non-ascertained sample (S2 Fig. (B)), mostly due to the abundance of monomorphic SNPs. In addition to differences in demographic processes, such as bottlenecks, differences in the extent and pattern of linkage disequilibrium may be the result of differences in the patterns of fine-scale recombination rate. We assessed the impact of fine-scale recombination events to differences in linkage disequilibrium patterns using a coalescent-based method [7]. Interestingly, we found that the southern African Bantu-speaking populations share proportionally more recombination hotspots with both Yoruba (YRI) and Europeans (CEU) than with the Ju\'hoansi (KHS) (Fig. 4, S6 Table), where a shared hotspot is identified as a region with greater than five times the background recombination rate within a 10kb window. The proportion of hotspots shared between southern Africans and both European (CEU) and Yoruba (YRI) samples was generally low (Fig. 4). Our empirical analyses indicate that few recombination hotspots are shared between southern Africans and the HapMap populations, with San being the most extreme. More results on recombination hotspots and the test of whether increased frequency of low frequency and monomorphic SNPs improves the power to detect recombination hotspots are detailed in S4 Text and S7 Table.

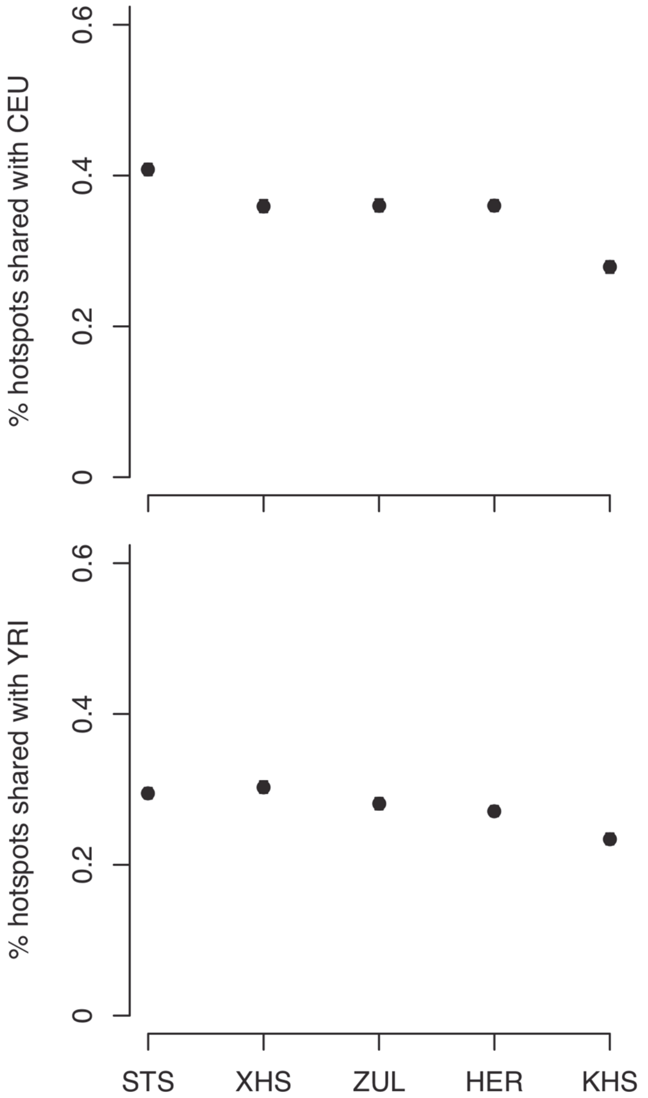

Fig 4. Proportion of shared recombination hotpots between the southern African and HapMap populations (CEU, YRI).

doi:10.1371/journal.pgen.1005052.g004

To assess the accuracy with which missing SNPs in southern African populations can be imputed using Yoruba (YRI) or European (CEU) reference populations, we removed SNPs, imputed them and checked for correctness in imputation (detail in S1 Text and S3 Text). Our results show that YRI appears to be useful for imputation, at least for some of the southern Bantu-speaking groups included in the study, namely Sotho/Tswana (STS), Zulu (ZUL), Herero (HER) and Xhosa (XHS), but less so for the San, for whom imputation accuracy is significantly lower than for other African populations (S3 Fig.). Xhosa (XHS) also had lower imputation accuracy, compared with other Bantu-speaking groups.

Unusual Differentiation in Allele Frequencies

We first developed an approach to select polymorphisms that exhibit large allele frequency differences between ancestral populations of Sandawe (SAW), Xhosa (XHS) and ‡Khomani (KHO) (see Materials and Methods). We constructed 3 different panels of AIMs [for Sandawe

(SAW), Xhosa (XHS) and ‡Khomani (KHO)], where selected SNPs have a certain level of admixture LD with each other and with at least 1MB spacing between adjacent genetic markers on a chromosome (Materials and Methods). This was to avoid linkage disequilibrium (LD) in the ancestral population. Such background LD could contribute noise (or bias) to the estimation of ancestral allele frequencies and locus-specific ancestry [43]. Thinning down the SNPs to a 1Mb spacing may result in a reduction in power to detect cases of deviation in ancestry or allele frequency differences that result from selection. Consequently, our strategy to detect regions of unusual differentiation between the admixed southern African populations and their source populations, and unusual deviation in local ancestry, is conservative. We evaluated whether there is an excess of common SNPs with large allele frequency differences (expressed as a $\chi 2$ (1 d.o.f.) statistic under a model (see Materials and Methods) of neutral genetic drift) between putative ancestral populations of each admixed southern African population [‡Khomani (KHO), Sandawe (SAW) and Xhosa (XHS) (Table 1 and S5 Fig.)]. An unusual extent of population differentiation can suggest the action of population-specific natural selection. We observed several SNPs within chromosomal regions (Table 1) for which the evidence of unusual population differentiation was genome-wide significant between the Sandawe (SAW) and Caucasian (CEU) populations (S5 Fig.), and a small number of SNPs (on chromosome 17q25.1 and 12q24.21) showed unusual genome-wide significant differentiation between SAW and its two other putative ancestral populations, Yoruba (YRI) and Ju\'hoansi (KHS) (S5 Fig.). Chromosome region 3p11 yielded (to) a genome-wide significance of unusual differentiation between the Xhosa (XHS) and Ju\'hoansi (KHS) (p = 9.5e-10, lowest p-value), and between ‡Khomani (KHO) and Ju\'hoansi (KHS) (p = 7.6e-09, lowest p-value). Furthermore, unusual allele frequency differences between the Yoruba (YRI) and Xhosa (XHS) were identified on chromosome 1q41. No significant signal of unusual allele frequency differences between Yoruba (YRI) and ‡Khomani (KHO) were observed, which may be explained by the fact that the Niger-Congo contribution to admixture in the Khoe-San groups, in particular the ‡Khomani (KHO) (Khoe-San population) occurred too recently for it to have a significant impact on their allele frequencies. All these identified candidate SNPs of unusual allele frequency differences lie in or near known genes (Table 1). Their biological functions in the GeneCards database [44], are putatively linked with diseases of high prevalence in southern Africa; their detailed annotations are presented in Table 1.

Local Ancestry in XHS, SAW and KHO

We selected the best proxy parental populations of Xhosa (XHS) based on a pool of Click-speaking and Bantu-speaking populations using PROXYANC [45]. Yoruba (YRI) and Ju\'hoansi (KHS) were chosen as best proxy ancestral populations for Xhosa (XHS). Similarly, among the populations in the study, Yoruba (YRI), European (CEU) and Ju\'hoansi (KHS) were chosen as best non-San, European and San proxy ancestral populations for both ‡Khomani (KHO) and Sandawe (SAW) (Materials and Methods). Using AIMs panels, LAMP-LD [46] was employed to estimate the distribution of genetic contributions of ancestry across the genome (Materials and Methods) to provide additional reassurance from our data that we obtain unbiased results in the absence of possible background LD. The average locus-specific Ju\'hoansi (KHS) and Yoruba (YRI) ancestry proportions across the Xhosa (XHS) samples were estimated to be 27% ± 3.1% and 73% ± 3.1% (mean ± SD), respectively. We obtained 12% ± 0.8%, 77% ± 1.1% and 11% ± 0.9% (mean ± SD) locus-specific Yoruba (YRI), Ju\'hoansi (KHS) and Caucasian (CEU) average ancestry contributions, respectively along the genome of the ‡Khomani (KHO). For the Sandawe (SAW), the locus-specific ancestry proportions were 12% ± 0.9%, 70% ± 0.7% and 18% ± 1.0% for Yoruba (YRI), Ju\'hoansi (KHS) and Caucasian (CEU) average ancestry, respectively. The

Table 1. List of SNPs whose unusual differentiation between pair-wise indigenous southern African populations are genome-wide significant.

CHR	SNP	A1/ A2	P values			Gene	Pathway	Associated Disease
**Yoruba and Xhosa**								
1q41	rs11118642	C/A	1.1e-13			HLX	Metabolic	Fryns syndrome, Hernia, acute myeloid leukemia
**Yoruba and Sandawe**								
17q25.1	rs2279053	C/A	5.5e-10			QRICH2	Metabolic	Drug metabolism other enzymes
12q24.21	rs4767374	C/A	1.3e-09			MED13L	Obesity/Transposition-of-Great-Arteries	Drug metabolism other enzymes
**European (CEU) and Sandawe**								
12p11	rs3816834	C/A	2.9e-08			ITPR2	Long term depression	Amyotrophic lateral Sclerosis, arrhythmia, rheumatism, Alzheimer's disease, hypertension, liver cancer, hepatitis b and pancreatitis
11q22.1	rs1943760	C/A	4.0e-09			PGR	Immune response MIF-JAB1 signalling, Oocyte meiosis	Thyroiditis, breast carcinoma, tumors, carcinoma ductal and Breast cancer
21q22.3	rs2839439	C/A	9.6e-09			C21orf121	Metabolic	Choroiditis and Down Syndrome
2p21	rs4588165	A/C	1.5e-09			CRIM1	MAPK signalling pathway	Neuronitis, ataxia and macular degeneration
6q22.2	rs2049923	C/A	9.3e-10			MARCKS	Fc gamma R-mediated phagocytosis	Hepatitis, malignant syringoma, bipolar disorder, brain disease, Alzheimer's disease, asthma, and colorectal cancer
7q21	rs4730838	C/A	2.5e-10			MAGI2	Tight junction	Ulcerative and Colitis-and-Crohn's-Disease
6q25.1	rs9384458	A/C	2.9e-08			ARID1B	Drug metabolism and other enzymes	Coffin-Siris and Syndrome
12p13.3	rs11062658	C/A	1.6e-09			PRMT8	Drug metabolism other enzymes	Malaria, peripheral primitive neuroectodermal tumor, primitive neuroectodermal tumor, and neuroectodermal tumors
6q23.2	rs9478984	A/C	3.5e-08			RPS12	Ribosome	Malaria, Carcinoma and Tuberculosis
3q21.2	rs1373606	A/C	2.9e-08			KALRN	Drug metabolism and other enzymes	Neuronitis Human Immunodeficiency Virus Infectious disease
4q34.3	rs1567475	C/A	1.3e-08			AGA	Glycan degradation	Influenza and Aspartylglucosaminuria
2q21.2	rs1561019	C/A	9.6e-09	0.23	0.04	LRP1B	Metabolic	Cholesterol Thyroiditis
9q31.2	rs7039618	C/A	9.6e-09	0.23	0.04	TMEM38B	Metabolic	Cleft Lip
7q31.1	rs2037048	C/A	2.9e-08	0.23	0.04	C7orf66	-	-
14q21	rs2054492	C/A	5.2e-08	0.23	0.04	PELI2	Metabolic	Ataxia
5q11.2	rs1075420	C/A	9.6e-09	0.23	0.04	MAP3K1	GnRH signaling	Breast Cancer
12q24.32	rs10773557	A/C	9.6e-09	0.23	0.04	TMEM132C	-	-
**Ju\'hoansi and ‡Khomani**								
3p11	rs4858960	A/C	7.6e-09	0.25	0.04	POU1F1	Metabolic	Combined Pituitary Hormone deficiency, growth hormone deficiency
**Ju\'hoansi and Sandawe**								

(*Continued*)

Table 1. (*Continued*)

CHR	SNP	A1/ A2	P values			Gene	Pathway	Associated Disease
14q21.1	rs10148725	C/A	1.8e-08	0.24	0.04	FBXO33	Metabolic	Osteoporosis
3p11	rs4858960	A/C	5.3e-10	0.24	0.04	POU1F1	Metabolic	Combined Pituitary Hormone deficiency, growth hormone deficiency
1q25	rs234654	C/A	1.8e-08	0.24	0.04	FAM129A	Metabolic	Carcinoma
3q28	rs260559	A/C	1.8e-08	0.24	0.04	TPRG1	Metabolic	Parkinson's Disease
14q13.2	rs10132268	C/A	1.8e-08	0.24	0.04	INSM2	Metabolic	Insulinoma
**Ju\’hoansi and Xhosa**								
3p11	rs4858960	A/C	9.5e-10	0.25	0.04	POU1F1	Metabolic	Combined Pituitary Hormone deficiency, growth hormone deficiency
3q13.32	rs1521293	C/A	3.2e-08	0.25	0.05	IGSF11	Metabolic	Carcinoma

We obtained the associated disease genes using the MalaCards Database, an integrate compendium for diseases and their annotations [44, 53].

doi:10.1371/journal.pgen.1005052.t001

above estimates of average locus-specific ancestry are all consistent with the related genome-wide average proportion estimates in the admixture analysis section, indicating that there is no evidence of systematic distortion in our local ancestry estimates. The plots of these average locus-specific ancestries of these admixed southern African populations, namely Xhosa (XHS), ‡Khomani (KHO) and Sandawe (SAW) are in S6 Fig.. In the next two sections, we examined signals of selection, consisting of unusual deficiency or excess of ancestry in the admixed southern Xhosa (XHS), Sandawe (SAW) and ‡Khomani (KHO) populations. Such regions in admixed populations have served in previous studies as signatures of natural selection that occurred after admixture [43, 47, 48, 49, 50, 51]. Here, we considered not only the regions of strong deviation from ancestry, but we also implemented an approach that is now incorporated in PROXYANC [45] to test for unusual deficiency or excess ancestry using the inferred locus-specific ancestry across the genomes of admixed populations. The loci showing unusual ancestry patterns, i.e. four standard deviations above (excess ancestry) or below (reduced ancestry) the genome-wide average, were identified as candidates of post-admixture natural selection (Materials and Methods).

Identification of Regions of Unusual Excess or Reduced Ancestry in the Xhosa (XHS) population

Examining the genome-wide distribution of ancestry in Xhosa (XHS), we detected the natural selection events post-admixture (Table 2). We identified a region on chromosome 3p11 (chr3: size: 17,184 (bp), p = 1.4e-10) with strongly reduced Ju\’hoansi (KHS) ancestry in Xhosa (XHS) (Table 2). This region yielded a genome-wide significance with an unusual difference of ancestry, suggesting a signal of selection after admixture. The SNP in the 3p11 region with the lowest p-value, rs4858960, is associated with *POU1F1*, which in turn interacts with five other genes [52], including *ETS1*, *NR3C1*, *JUN*, *NR1I3* and *MED1*. These genes are known to play a role in a metabolic pathway that positively affects growth traits and hormone deficiency [53]. Furthermore, the 3p11 region showed strong differences in allele frequencies between Xhosa (XHS) and Ju\’hoansi (KHS) (p = 9.5e-10) (Table 1). Since San and Khoe-San communities have undergone a sharp population decline in their history, this differentiation suggests an

Table 2. Four regions showing excess of YRI ancestry and three regions of reduced CEU and KHS ancestry in ‡Khomani.

Region	SNPs	Position	Size (bp)	Excess Ancestry	Lowest P value	Genes	Pathway	Associated Disease
12q24.1	4	112,842,994–112,856,642	13,649	YRI	4.2e-9	RPL6,RPL11,RPS3, RPS15A,RPL4,RPL7	Ribosome	Malaria, Noonan syndrome, t-cell leukemia, colorectal cancer, gastric cancer, carcinoma, lupus erythematosus
13q14.3	2	58,513,521–58,515,045	1,525	YRI	3.1e-08	IHNRNPA1,HNRNPA1L2,	Spliceosome	Malaria
18p11.23	12	6,729,821–6,915,715	185,895	YRI	2.2e-08	LAMA1,ARHGAP28, ITGA1,ITGA2,C3,LAMB1, PLAT	Signalling by Rho GTPases,Signal Transduction, Pathways in cancer,Focal adhesion	Meningioma, Lung cancer, Congenital muscular dystrophy, Neuromuscula, Alzheimer's and Hirschsprung's diseases
18p11.31	18	5,954,705–6,414,910	460,206	YRI	2.9e-08	L3MBTL4,LOC100130480	-	Parkinson's disease, Breast cancer
**Region**	**SNPs**	**Position**	**Size (bp)**	**Deficient Ancestry**	**Lowest P value**	**Genes**	**Pathway**	**Associated Disease**
12p13.31	1	7,864,050–7,870,155	6,106	KHS	3.7e-08	DPPA3,IPO5	-	Seminoma, Testicular germ cell tumor, Teratocarcinoma, Germ cell tumor,Carcinoma
14q13.2	14	36,007,558–36,278,510	270,953	KHS,CEU	3.4.e-09	GARNL1,BRMS1L, RALGAPA1,NFKBIA, INSM2,NFKB1,PTCSC3, KIAA0391,RELA,CHUK, IKBKB,IKBKG,TCF3,MYC, ZSCAN1,SAP30,BRMS1, RBBP4,RBBP7,ING2	Toll-Like Receptors Pathway	Tuberous sclerosis, Prader-Willi syndrome, Breast cancer, Lung cancer, Tumors inflammation, Leukemia T-cell, Diabetes mellitus, Parkinson's disease
							Molecular Mechanisms of Cancer	
							NF-kappa B Activation by Viruses, Itk and Tcr Signalling	
14q13.3	7	36,985,602–36,990,354	4,753	KHS,CEU	1.1e-08	NKX2–1,NKX2–8, PTCSC3,SFTA3, CCDC59,NCK1,MAPK1, NCOA2,RARA	Cell adhesion Tight junctions	Chorea benign Hereditary, Hepatocellular carcinoma, Lung cancer, Adenocarcinoma lung

We obtained the associated disease genes using the MalaCards database [44, 53] (Materials and Methods).

doi:10.1371/journal.pgen.1005052.t002

environmental pressure that the San ancestors of the Xhosa (XHS) may have experienced before population admixture, and we speculate a possible adaptation of Xhosa (XHS) to the local environment. Mutations in the *POU1F1*/*PIT1* gene, a pituitary-specific transcription factor, affect the development and function of the anterior pituitary and lead to combined pituitary hormone deficiency [53].

Identification of Regions of Highly Unusual Excessive or Reduced Ancestry in the Sandawe (SAW) and ‡Khomani (KHO) Populations

In spite of slight predominance of Ju\'hoansi (KHS), San ancestry in ‡Khomani (KHO) compared to Sandawe (SAW), and European (CEU) related ancestry in Sandawe (SAW) compared to ‡Khomani (KHO), consistent with previous findings [16, 26, 34, 40], our results from both

Table 3. Three regions showing excess of YRI ancestry and other three showing deficiency of CEU and KHS ancestry in Sandawe.

Region	SNPs	Position	Size (bp)	Excess Ancestry	Lowest P value	Genes	Pathway	Associated Disease
12q24.1	4	112,842,994–112,856,642	13,649	YRI	1.4e-10	RPL6,RPL11,RPS3, RPS15A,RPL4,RPL7	Ribosome	Malaria, Noonan syndrome, t-cell leukemia, colorectal cancer, gastric cancer, carcinoma, lupus erythematosus
18p11.31	30	5,954,705–6,414,910	460,206	YRI	1.9e-13	LOC645355,L3MBTL4, MIR3976,ARHGAP28, LOC100130480	Signalling by Rho GTPases, Signal Transduction	Benign meningioma, Meningioma, Parkinson's disease, Hamartoma, Retinitis, Acute myeloid leukemia
18p11.23	13	6,941,743–7,117,813	176,071	YRI	2.0e-12	LAMA1	Cell adhesion Endothelial cell contacts by non-junctional mechanisms and Cytoskeleton remodelling Integrin outside-in signalling	Muscular dystrophy, Myopia, Choriocarcinoma, Congenital muscular dystrophy, Alport syndrome, Hirschsprung and Alzheimer's disease
**Region**	**SNPs**	**Position**	**Size (bp)**	**Reduced Ancestry**	**Lowest P value**	**Genes**	**Pathway**	**Associated Disease**
12p13.31	1	7,864,050–7,870,155	6,106	KHS,CEU	3.3e-08	DPPA3, IPO5	-	Seminoma, Testicular germ cell tumor, Teratocarcinoma, Germ cell tumor, Carcinoma
14q13.2	11	36,007,558–36,278,510	270,953	KHS,CEU	1.4.e-10	GARNL1,BRMS1L, RALGAPA1,NFKBIA, INSM2,NFKB1,PTCSC3, KIAA0391,RELA,CHUK, IKBKB,IKBKG,TCF3, MYC,ZSCAN1,SAP30, BRMS1,RBBP4,RBBP7, ING2	Toll-Like Receptors Pathway	Tuberous sclerosis, Prader-Willi syndrome, Breast cancer, Lung cancer, Tumours inflammation, Leukemia T-cell, Diabetes mellitus, Parkinson's disease
							Molecular Mechanisms of Cancer	
							NF-kappa B Activation by Viruses	
							ITK and TCR Signalling	
							RANK Pathway	
14q13.3	5	36,985,602–36,990,354	4,753	KHS,CEU	2.3e-08	NKX2–1,NKX2–8, PTCSC3,SFTA3, CCDC59,NCK1,MAPK1, NCOA2,RARA	Cell adhesion Tight junctions	Chorea (Benign Hereditary), Hepatocellular carcinoma, Lung cancer, Adenocarcinoma lung

We obtained the associated disease genes using the MalaCards Database [44, 53] (Materials and Methods).

doi:10.1371/journal.pgen.1005052.t003

admixture (Fig. 1) and locus-specific ancestry analyses (S6 Fig.) have shown a potential ancestral link between the admixed Sandawe (SAW) and ‡Khomani (KHO). Three chromosomal regions (12q24.1, 18p11.31 and 18p11.2), each within several SNPs with moderate and significant p-values, appear with excess of Yoruba (YRI) ancestry in both Sandawe (SAW) and ‡Khomani (KHO); an additional region (13q14.3) was also identified as an excess of Yoruba (YRI) ancestry in ‡Khomani (KHO), (Tables 2 and 3). These four candidate regions (Tables 2 and 3) showed strong unusual difference of ancestral contributions ($p < 1.0$ e-08, chi2 test),

Table 4. Number of known copy number polymorphisms (of a total of 1130 autosomal CNPs) that are polymorphic in each analysis panel.

	Number of polymorphic CNPs	Proportion of CNPs polymorphic
**CEU**	577	0.63
**YRI**	837	0.92
**STS**	486	0.50
**XHS**	636	0.67
**ZUL**	561	0.57
**HER**	482	0.49
**KHS**	338	0.38

doi:10.1371/journal.pgen.1005052.t004

and have been associated with various important diseases, including malaria, T-cell leukemia, congenital muscular dystrophy, Noonan syndrome [53], and others listed in Tables 2 and 3. That some genes in these regions are associated with ‡Khomani (KHO)- and Sandawe (SAW)-specific high-risk diseases (such as malaria) [53], suggests a functional role these disease-related genes (or other genetic elements in these regions) might have played in their migration and particularly local adaptation due to such selective pressure resulting from shared gene-culture co-evolution and cultural practices in Bantu-speaking and Click-speaking populations. Overall, in the results of genome-wide allele frequency differences between Yoruba (YRI) and these two admixed populations (Tables 1, 2 and 3), only the 12q24.1 region was replicated significantly between Yoruba (YRI) and Sandawe (SAW). This may indicate different environmental pressures that the ‡Khomani (KHO) and Sandawe (SAW) experienced post-population-admixture.

We observed two other regions (12p13.31 and 14q13.2–14q13.3), with significant difference (Tables 2 and 3) of ancestry ($p < 4.8e\text{-}08$) showing a strong relative reduction of Caucasian (CEU) and Ju\'hoansi (KHS) ancestry in both ‡Khomani (KHO) and Sandawe (SAW). These regions were also identified as candidates of the natural selection after admixture (Tables 2 and 3). Importantly, these two regions (Tables 2 and 3) are also associated with some important diseases such as breast cancer, lung cancer, tumour inflammation, diabetes mellitus, Parkinson's and other diseases [44, 53], Although these regions have been associated with diseases, there is no indication of whether this points to any mechanistic association. However, it is tempting to speculate that factors such as food, pathogens, and life style, could also be responsible for such reduction in ancestry and may therefore play a role

Copy Number Variation

Our approach to analyzing copy number variation in southern African populations involved the detection of known copy number polymorphisms (CNPs) using a Gaussian mixture model, and the identification of potential novel copy number variants (CNVs) using a Hidden Markov Model (HMM) (S5 Text). The number of CNPs (S5 Text) in Yoruba (YRI) is greater than that found in the European (CEU) and the southern African populations (Table 4). The former is probably the result of bottlenecks in non-Africans and subsequent loss of CNPs of low frequency [54, 55, 56], whereas the latter is likely the result of ascertainment bias. Given that CNP probes were ascertained in HapMap populations (including Yoruba (YRI)), lower levels of CNP diversity for populations that are divergent from ascertained populations is expected. However, southern African populations, which are approximately matched for sample size, show marked differences in the distribution of the number of CNPs, particularly in the San (Ju\'hoansi (KHS)) with fewer CNPs than other southern African populations (Table 4). Distributions of derived allele frequencies of CNPs suggest higher purifying selection on duplications (S7 Fig.). In contrast,

however, there appears to be little difference in the degree of purifying selection on duplications and deletions in novel CNVs detected with the HMM (S7 Fig. (A)). We detected a total of 1873 CNVs (Table 5), of which 1231 were deletions. Only 137 of the CNVs were singletons, with 87 deletions and 50 duplications (Table 6). A total of 397 were novel with respect to the Database of Genomic Variants [55, 56, 57, 58]. At least 157 of these were unique CNVs, which occurred in only one population. The number of CNVs per individual is generally similar between populations (S7 Fig. (B)), except San which had significantly fewer deletions than other populations [e.g. Herero (HER) vs Ju\'hoansi (KHS)]: Student's T-test, $t_{20} = 22.4$, $P = 1.3e\text{-}15$). Furthermore, distributions of derived allele frequencies of CNPs suggest purifying selection on duplications (S7 Fig. (A)). In contrast, however, there appears to be little difference in the degree of purifying selection on duplications and deletions in novel CNVs detected with the HMM (S7 Fig. (A)).

Discussion

In this study, we have conducted a systematic population genomics survey and investigated demographic histories of indigenous southern African populations, making it possible to address questions about the signature of selection prior to and following purported ancient admixture events. Consistent with previous studies [16, 26, 33, 34, 35, 39, 40], we demonstrated stratification among indigenous southern African populations. Both the geographic distribution of genetic variations and the population structure, suggested a complex human population history generally within the African continent, and specifically in southern and eastern Africa. Incorporating the data from other Click-speaking populations from previous studies [16, 26, 33, 34, 39, 40] together with that from our 25 Ju\'hoansi (KHS) subjects, it was possible to investigate the relationship between Click-speaking and southern Bantu-speaking populations thought to represent an early diverging branch of modern humans.

The admixture analyses, particularly that of southern African populations, lends support of gene flow between San and Niger-Congo-speaking populations due to their contact following migrations of Bantu-speaking populations across the continent [17, 18, 26, 27, 33, 34, 35]. Consistent with previous studies [16, 26, 33, 34, 39, 40], our admixture (Fig. 1) and tree-mix analyses (S1 Fig.) suggested a division between south-west (San) and south-east (Khoe-San mostly

Table 5. Copy number variants shared among study populations and with previously reported structural variants.

	CNVs	Novel CNVs*
HapMap & Southern African	279	39
African only	315	51
Southern African only	323	51
Southern African Bantu	429	61
**Total**	1873	397
**Private CNVs**		
CEU	210	65
YRI	145	40
STS	37	11
XHS	69	14
ZUL	32	8
HER	28	11
KHS	47	8

*compared to Database of Genomic Variants

doi:10.1371/journal.pgen.1005052.t005

Table 6. Number of singleton copy number variants (CNVs) in each population.

Population	Singletons	Deletions	Duplications
CEU	63	63	0
YRI	24	0	24
STS	8	8	0
XHS	23	14	9
ZUL	12	2	10
HER	2	0	2
KHS	5	0	5
	**137**	**87**	**5**

doi:10.1371/journal.pgen.1005052.t006

admixed) populations. Our findings confirm an ancient link between San and some eastern African populations, including Sandawe, consistent with previous findings [16, 26, 35, 34, 39, 40]. The Eurasian ancestral components in south-east Khoe-San and some eastern Bantu speaking populations (such as Sandawe, Hadza) may be a consequence of an early Eurasian genetic contribution into Africa [16, 28, 35], Furthermore, the f-3 statistic test (S3 Table) confirms southern Bantu speaking populations, in particular Xhosa (XHS) to be two-way admixed, and both ‡Khomani (KHO) and Sandawe (SAW) are at least three-way admixed. The San (KHS) exhibit higher levels of homozygosity (S9 Table), increased relatedness (S9 Table) and higher proportions of monomorphic SNPs (S8 Table) than other African populations. However, we have shown that ascertainment of markers in a divergent population results in a reduction of diversity in the genotyped population, probably the result of polymorphisms arising after the divergence of the ascertained and genotyped populations, and the loss of polymorphisms in the genotyped population through fixation. Improved statistical models are therefore needed for the comparison of populations that have varying degrees of divergence from the population in which markers were ascertained.

Our copy number analysis included identification of both known CNPs, which are copy number loci previously identified in HapMap populations [55, 56, 58], and putatively novel CNVs. CNPs are highly ascertained, since they have been selected to be polymorphic and segregating at allele frequencies > 1% in HapMap populations [56]. CNVs, however, are less ascertained and should have more similar levels of polymorphisms in all of the studied populations [55]. In the case of CNVs, deletions are observed more frequently than duplications. This appears to be inconsistent with the proposal that deletions are under stronger purifying selection [58, 59, 60], which has also been inferred previously based on a lower degree of overlap between deletions and both genomic regions [59], and disease-related genes [59]. However, the disparity in the number of deletion and duplication CNVs probably reflects the relative difficulty of detecting the latter, due to a smaller relative change in copy number (3:2 versus 2:1) [59], rather than stronger purifying selection on duplications. In the southern African data, deletions and duplications have similar distributions to that of derived allele frequencies for CNVs, suggesting little difference in the relative degree of purifying selection. The number of deletion CNVs per individual differs markedly between the San (KHS) and other African populations. This may be an effect of sample size; however Herero (HER), with a similar sample size to San (KHS) for copy number calling, have no reduction in the number of deletions. In addition, copy number variants called for the Zulu (ZUL) panel with only 20 samples, were more than 99.9% concordant at normal, and 81.6% concordant at abnormal copy number regions, with those called in conjunction with other Bantu populations. Alternatively, some hybridization probes may have lower intensities in the San (KHS) due to probe-target mismatch

mutations. However, such probe effects are likely to cause increased numbers of deletions in the San (KHS). Finally, population demographic and selective effects may cause differences in the number of deletion CNVs. In summary, copy number results suggest San (KHS) to be unique, although they should ideally be validated using trios, as shown previously [55, 56].

Haplotype blocks show very similar patterns of linkage disequilibrium between African populations, with this collective group having substantially shorter haplotype blocks, and less linkage disequilibrium, than Non-African populations. For instance, patterns of linkage disequilibrium surrounding the lactose tolerance (LCT) gene, known to have undergone a selective sweep in Europeans [7], have strong levels of linkage disequilibrium in Europeans, yet not in southern African populations (S2 Fig. and S4 Fig.). Khoe-San, however, appear to have increased levels of linkage disequilibrium associated with LCT than the other African populations [particularly the Sotho/Tswana (STS) and Zulu (ZUL); S2 Fig.]. This may be due to a weak selective sweep or the result of gene admixture with the San (KHS), a pastoral group from Namibia known to be lactose tolerant [29].

In addition, it was particularly interesting to examine the signature of selection in the indigenous and admixed southern African populations, including ‡Khomani (KHO), Xhosa (XHS) and Sandawe (SAW) due to the high mortality of the San population, historically. Following the recommendation of Bhatia et al. [61], we additionally implemented two strategies to detect possible evidence of population-specific natural selection in southern African populations. The first strategy, involved evaluating whether there is an excess of common SNPs with large allele frequency differences between admixed southern African populations, including ‡Khomani (KHO), Sandawe (SAW) and Xhosa (XHS) and their purported parental populations. The power of this analysis was based on an approach we developed to select three panels of 502 SNPs with at least 1MB spacing between adjacent genetic markers on each individual chromosome. Several SNPs on chromosomal regions for which there is evidence of unusual population differentiation between Sandawe (SAW) and Caucasians (CEU), are displayed in Table 1. Importantly, most of the signals of selection identified through this strategy are linked with specific high-risk diseases such as malaria, influenza, tuberculosis, and AIDs/HIV, which have a high prevalence in southern African populations (e.g. in the Sandawe, ‡Khomani and Xhosa populations) (Table 1). The allele frequency differences between southern African populations (including some putative parental populations) follow the null distribution predicted by neutral drift as a consequence of the recent origin of southern African population structure. This may yield a risk of false positive associations due to population stratification in disease association studies, despite the fact that there are differences between southern African populations [62].

The second strategy to detect possible evidence of population-specific post-admixture selection involved a signal of unusual excess or deficiency of ancestry in the admixed southern African populations [‡Khomani (KHO), Sandawe (SAW) and Xhosa (XHS)]. The recent studies by Bhatia et al. [61, 63] showed that loci with significant deviation in local ancestry (from the genome-wide average) may due to insufficient correction for multiple hypothesis testing and/or due to possible systematic errors in local ancestry inference. We have employed the minor allele frequencies from the correct proxy ancestral populations of the admixed population to correct for possible systematic errors on the inferred local ancestry that may lead to false positive deviations in local ancestry. Moreover our study did not only rely on the deviation (more than 4.0 standard deviations) in local ancestry from the genome-wide average; we additionally used the distribution of difference in locus-specific ancestry along the genome admixed population to evaluate the genomic regions showing unusual excessive or reduced ancestry which are likely to be signatures of natural selection after admixture [43, 48, 49, 50, 51].

Several recent studies have detected excessive or reduced ancestry contributions in admixed populations as signals of post-admixture selection, using reference ancestral parental

populations [43, 48, 49, 50, 51]. Our study used selected best proxy ancestral populations and AIMs panels for our admixed southern African populations, and we extended previous approaches to test for unusually increased or decreased ancestry contribution along the genome. We identified three and four regions showing a significant excess of Yoruba (YRI) ancestry in Sandawe (SAW) and ‡Khomani (KHO), respectively (Tables 2 and 3). Three other regions showed unusually reduced Caucasian (CEU) and San (KHS) ancestry in both ‡Khomani (KHO) and Sandawe (SAW) (Tables 2 and 3). Since some of the genes in these regions are linked with specific high-risk diseases such as malaria in the ‡Khomani (KHO) and Sandawe (SAW), as has also been noted in the recent study by Gurdasani et al. [35], it is plausible that these disease-related genes might have played a role in population adaptation historically. Among the identified genomic regions, the 12q24.1 region was found in both strategies for detecting signals of natural selection, supporting evidence of environmental pressures that the ‡Khomani (KHO) and Sandawe (SAW) experienced. Furthermore, two other candidate regions pointing to natural selection were identified in both ‡Khomani (KHO) and Sandawe (SAW), showing strong deficiency of European and San ancestry components, and also an unusual population differentiation in these regions. These two regions are also linked with some important diseases such as breast cancer, lung cancer, inflammation, diabetes mellitus and Parkinson's disease [53], which are known to occur at a relatively higher prevalence in European populations, when compared to indigenous southern African populations [59].

African, and particularly southern and eastern African populations, face a heavy burden of diseases including HIV/AIDs, tuberculosis and malaria, and a growing burden of non-communicable diseases [17]. Of note, all the reported regions with signals of selection are in admixture LD and with significant deviation in average local ancestry (or unusual difference in allele frequency). In addition, our constructed AIMs panels for southern and eastern admixed populations may potentially be utilized for further admixture mapping studies in these populations. Nevertheless, further investigations are required to reveal the targets and agents of selection that have played important roles in shaping the admixed gene pool of these southern and eastern African admixed populations. With extensive admixture, both between none-San and San populations, and between African and non-African populations, southern and eastern African populations have a great potential for the identification of genes which determine susceptibility to both communicable and non-communicable diseases and to understand the African genetic variations with response to drugs/treatment variability.

The southern Bantu and Khoe-San populations are 'admixed' and future genome-wide studies will need to correct for this stratification or may need to use the locus-specific ancestry to increase power in association studies. Admixture mapping in the African-American and some other three-way admixed populations (such as Latinos, Puerto) has been successful for some disease traits [43, 51]. Since the admixed southern African populations have similar admixture proportions to admixed American populations, we hypothesize that admixture mapping would likely be a successful approach in many southern Bantu and Khoe-San cohorts, and particularly in the Xhosa, ‡Khomani and Sandawe.

A large proportion of the currently active genomic studies being conducted as part of the recently launched H3Africa programme (H3Africa, http://h3africa.org/) and the more recently described African Genome Variation Project [35], involve genome wide association studies [64]. A significant number of these studies involve large collections of sub-Saharan African subjects, and would benefit from this knowledge.

Materials and Methods

Ethics Statement

This study, investigating the genomic structure of indigenous southern African populations, was approved by the Research Ethics Committees of the University of Cape Town, and Witwatersrand University (REC Ref 305/2009 for the Project: Genome Wide Microarray Analysis of southern African Human Populations [65, 66].

Genetic Marker Selection: Relationship between Population Differentiation and Admixture Linkage Disequilibrium

Consider a pair of populations *k* and *l* from a pool of K ancestral populations of an admixed population and assume that the minor allele frequencies at SNPs *i* and *j* are greater than 0.005. Similar to Glaubitz et al. [67], we defined the admixture linkage disequilibrium as

$$L_{ij} = mL_{ij}^{k} + (1-m)L_{ij}^{l} + m(1-m)\delta_i^{kl} \times \delta_j^{kl} \qquad \text{(a)}$$

Where m is the ancestral proportion, δ_i and δ_j are differences in allele frequency at SNPs *i* and *j* in population *k* and *l*, respectively. Assuming for each pair of SNPs *i* and j there is no linkage disequilibrium in ancestral populations, it thus follows,

$$L_{ij} = m(1-m)\delta_i^{kl} \times \delta_j^{kl} \qquad \text{(b)}$$

$$1 = \frac{m(1-m)\delta_i^{kl} \times \delta_j^{kl}}{L_{ij}} \qquad \text{(c)}$$

At a given pair of SNPs *i* and *j* in the admixed population, Equation (c) establishes a relationship between the observed linkage disequilibrium L_{ij} in a recently admixed population and ancestral population differentiation. One can expected the ratio (part 2) in Equation c to be closer to 1 when the two reference ancestral populations contributed to the admixture of the related admixed population. Equation (c) is a total ancestry content (AC) at a pair of SNPs *i* and *j*. *Let I_{ij} denote the ration in Equation* c, assuming a uniform ancestral proportion, and summing Equation (c) over all possible pairs of proxy ancestral populations, we can obtain the ancestry informativeness I_{ij} of each pair of SNPs *i* and *j* as follows,

$$I_{ij} = \frac{1}{4\mathrm{K}} \sum_{k \neq l} \frac{\delta_i^{kl} \times \delta_j^{kl}}{L_{ij}}$$

Let M be the total number of SNPs. For $i \in \{1,\ldots, M\}$, let N_i be the total number of pair-wise LD j with i, where $j \neq i, \forall\, j \in \{1,\ldots, M\}$ within SNP *i*, we obtain the ancestry informativeness at SNP *i* as a weighted sum of I_{ij},

$$I_i = \sum_{j=1}^{N_i} \frac{I_{ij}}{\sqrt{M}}.$$

We applied this method to construct the AIMs panel for Xhosa, ‡Khomani and Sandawe. This approach of selecting ancestry informative markers (AIMs) is implemented in the PROXYANC program (http://web.cbio.uct.ac.za/proxyanc/).

Screening for Close Relatives and Admixture Analysis

We estimated the pair-wise genome-wide level of relatedness using a previously described relatedness statistic [67] applied to a random selection of 2500 putatively unlinked SNP markers with minor allele frequencies between 0.3 and 0.5. These SNPs were randomly selected across each chromosome, with a minimum spacing of 1 MB, to prevent inclusion of SNPs in strong linkage disequilibrium, which would violate the assumption of marker independence. Principal Component Analysis (PCA) was performed, using EIGENSOFT [68], on the combined HapMap3, HGDP, other African data from [26, 34, 39, 40] and southern African genotypes, which included a total of 50K SNPs shared between these different panels. In addition to the PCA analysis, an F_{ST} matrix using the smartpca program was generated. Admixture analysis [68, 69] was performed on combined panels based on 900K SNPs using the ADMIXTURE program [69]. To evaluate the genetic relationships among the above populations, we used the TreeMix software [40] to infer the structure of a graph from genome-wide allele frequency data and a Gaussian approximation to genetic drift. Furthermore, to identify some aspects of ancestry not captured by the tree, we also examined the residuals of the model's fit and sequentially added the migration events to the tree. We also used copy number variants as a population marker in an additional population structure analysis, but only for HapMap3 and southern African samples for which the intensity data (CEL files) necessary for copy number calling were publicly available. Copy number variants, detected with a Hidden Markov model that identifies novel copy number variation [55], were preferred over previously described copy number polymorphisms, since these are affected to a lesser extent by ascertainment bias. We randomly selected a total of 2869 copy number variable positions, corresponding to 1 marker every 1Mb, across all chromosomes and specified copy number alleles as either a deletion, normal or duplicated state dependent on the copy number state called in the Birdseye algorithm [55]. We only selected simple copy number variants consisting of either a deletion or duplication, but not both.

Relationship between Geographic and Genetic Distance

Here, we used all available southern African population data, including HER, SAN, XHS, XHS, LWK, BUS, ZUL, SAW, a Niger-Congo-speaking population (YRI) and a non-African population, which included CEU. We made use of the Haversine formula to compute the geographic distance (in kilometre) between pairwise populations based on great circle distances using the way points between continents. The way-points used are Egypt (29.998392, 30.999751) and Turkey (41.015472, 27.986336). Thus, we computed the correlation between F_{ST} and Geographic distance using a linear regression equation as

$$F_{ST} = 1.298 \times 10^{-5} \times \text{Geographic distance} + 1.709 \times 10^{-2}$$

We analysed the scatter plot of the relationship between F_{ST} and geographic distance. To address this, we computed the perpendicular distance between each point and the regression line. This enabled us to define outliers as points whose distance to the regression line is greater than or equal to 0.05 units.

Unusual Difference in Allele Frequency

To minimize deviation from the normality assumption, SNPs with minor allele frequencies < 0.05 are excluded. Thus, at a given locus i, the difference$(p_i^k - p_i^l)$ between observed variant allele frequencies of two populations, k and l, can be approximated as a normal

distribution under neutral drift with mean 0 and variance [60]

$$p(1-p)\left(2F_{ST}+\frac{1}{N_k}+\frac{1}{N_l}\right), \quad \text{(d)}$$

Where F_{ST} is the genetic distance between the population k and l. To avoid overestimating the degree of differentiation at single SNPs due to sample size difference, we used the estimator of F_{ST} in by Bhatia et al (63). N_k and N_l are total variant allele counts in each population, and p is the ancestral allele frequency that is commonly approximated as the average of the two observed variant allele frequencies. Similar to [60], we test unusual difference in allele frequency U_{kl} from population k and l as follows t

$$U^1_{kl}=\frac{(p^k_i-p^l_i)^2}{p(1-p)\left(2F_{ST}+\frac{1}{N_k}+\frac{1}{N_l}\right)}, \quad \text{(e)}$$

$$U^2_{kl}=\frac{(p^k_i-p^l_i)^2}{p(1-p)}. \quad \text{(f)}$$

Equations e and f are the χ^2 distributed with 1 degree of freedom (d.o.f), and can be applied to unrelated (Equation b) and related samples (Equation c), respectively. An excess of large values of the χ^2 statistic indicate deviations from the null model equation (Equation e and f), suggesting the action of natural selection [60]. We applied this method to the data from the Xhosa population using Ju\'hoansi and Yoruba as ancestral populations. We also applied this method to KHO and SAW using KHS, CEU and YRI populations. All gene annotations and associated diseases were obtained using both the GeneCards and MalaCards databases [44, 53].

Locus-specific Ancestry Inference

We used LAMP-LD to infer locus-specific ancestry in admixed populations [46]. The model in LAMP-LD leverages the structure of linkage disequilibrium in the proxy ancestral populations. LAMP-LD achieved highest accuracy in both simulation and real data in the study of Puerto Rico and Mexico populations [43]. Here, we applied LAMP-LD to infer local ancestry in three potential southern African populations, including KHO, XHS and SAW. Following the population structure result and the proxy ancestry selection approach developed in PROXYANC [45], YRI, KHS and CEU was selected as reference ancestral populations from a pool of Bantu-speaking, Click-speaking and European populations, respectively. We obtained phased haplotype data by running Beagle software [70] on KHS, CEU and YRI data. To estimate the distribution of genetic contributions of ancestries to XHS across the genome, we used haplotypes of 80 YRI and 80 KHS. In addition, the haplotypes of 80 YRI, 80 CEU and 24 KHS were used to compute the locus-specific genetic contributions to KHO and SAW using the AIMs panel.

Estimating Excess or Deficiency of Ancestry

Admixed populations provide special opportunities for investigating recent selection. Prior to admixing, the ancestral populations have been isolated geographically, and their genomes may have evolved in distinct environments. Migration of previously isolated populations may have brought individuals of the ancestral populations into an unusual environment, and may consequently introduce life-style changes or changes in pathogens they are exposed to. This type of selection may differ from that faced by stationary populations, for which the local environmental changes may occur gradually, allowing for rare advantageous alleles to increase in frequency [43]. Here, we adopted an approach to detect ancestral signatures of selection by looking in an

admixed population for genomic regions that exhibit unusually large deviations in ancestry proportions compared with what is typically observed elsewhere in the genome.

Given the genome-wide ancestral proportions, α_k, from ancestral populations $k \in \{1, \ldots, K\}$ in N samples of an admixed population, let $\varphi_k^{i,m}$ be the estimated locus-specific ancestry of individual i at genetic marker $m \in \{1, \ldots, M\}$, from the kth ancestral population. We computed the deficiency or excess of ancestry, at each SNP using the estimated admixture proportion as a baseline. We thus define the deficiency/excess of ancestry from ancestral population k at marker m as,

$$\delta_k^m = \left(\frac{1}{N}\sum \varphi_k^{i,m}\right) - \alpha_k = \bar{\varphi}_k^m - \alpha_k$$

where $\bar{\varphi}_k^m$ is the average locus-specific ancestry at SNP m. δ_k^m can be approximated as a normal distribution under neutral drift with mean 0 and empirical variance, derived from the distribution of $\varphi_k^{i,m}$ values among the N individuals [43, 51]. We can fit a chi-square on $\varphi_k^{i,m}$ as follows,

$$Z_k^m = \frac{(\delta_k^m)^2}{var(\varphi_k^{i,m})}$$

is a χ^2 with 1 degree of freedom. A large value of the chi2 statistic indicates deviations from the null model and 4 standard deviations above (excess ancestry) or below (deficiency ancestry) the genome-wide average, suggests the action of natural selection post-admixture [51]. Summing-up the equation above over all SNPs assigned to a gene, we obtain the deficiency/excess of ancestry at the gene level. This allows us to assess the statistical significance of a deficiency/excess of ancestry at the SNP and gene level. To assess unusual difference in deficiency/excess of ancestry between a pair of ancestral populations given SNP $m \in \{1, \ldots, M\}$ within a gene, we compute

$$\tilde{t}_{kl} = \sum \left(\frac{(\delta_k^m - \delta_l^m)^2}{\sqrt{[var(\varphi_k^{i,m}) + var(\varphi_l^{i,m})]/N}}\right)$$

Which is a two-sample t-statistic with M – 2 degrees of freedom, assuming equal sample size N. For a pair of populations, $k \neq l \in \{1, \ldots, K\}$, we compute the overall unusual difference in a deficiency/excess of ancestry,

$$\tilde{t} = \sum\sum \left(\frac{(\delta_k^m - \delta_l^m)^2}{\sqrt{[var(\varphi_k^{i,m}) + var(\varphi_l^{i,m})]/N}}\right)$$

Enrichment Analysis of Scans for Selection

In order to summarize the types of loci and explore the potential adaptive genetic architecture implicated by our genome-wide selection scans, we identified all protein coding genes within 40 kb downstream or upstream of SNPs showing signatures of selection. To achieve this, we downloaded genomic coordinates for all genes from the NCBI ftp-server (ftp://ftp.ncbi.nih.gov/), retaining only entries for the human reference sequence and protein-coding genes. We updated genomic coordinates to the latest assembly using the Lift-Over tool on GALAXY (https://main.g2.bx.psu.edu/). We obtained the genomic predicted human genes from the GeneCard database [44]. We investigate the roles of genes and cells in disease processes using the MalaCard database [44; 53].

Supporting Information

S1 Fig. (A-B) Maximum likelihood tree of indigenous southern Africa populations, including a proxy European ancestral population for southern Africa populations. (C) Residual fit from the maximum likelihood tree is plotted and the standard error of the entries in the covariance matrix is represented ten times on the scale bar.
(PNG)

S2 Fig. (A) Decay of linkage disequilibrium with physical distance for simulated data in which SNPs were not ascertained, ascertained in the genotyped (focal) population, or ascertained in a divergent population, where τ is the time of population divergence (see Methods). (B) Frequency spectra in the genotyped population, or in a divergent population, showing the frequency spectra to differ when SNPs are ascertained in a divergent population.
(TIFF)

S3 Fig. Imputation Accuracy of southern African genotypes using CEU or YRI haplotypes from 1000 Genomes Project.
(TIFF)

S4 Fig. Power to detect a simulated recombination hotspot (at 250kb) after a population bottleneck of different sizes (0.5*Ne; 0.7*Ne) at times τ = 0.00625, 0.06875 and 0,025 when markers are ascertained in the genotyped (focal) population, or in a divergent population.
(TIFF)

S5 Fig. Significant unusual differentiation in allele frequency between: (A) Ju\'hoansi and Sandawe, (B) Ju\'hoansi and Xhosa (C) Yoruba and Xhosa, (D) Ju\'hoansi and ‡Khomani (E) Yoruba and Sandawe (F) European and Sandawe.
(TIFF)

S6 Fig. Average locus-specific ancestries of these admixed southern African populations. Plot (A-C) consist of 47, 864 randomly selected SNPs along the entire genome. (A) Ancestry segments in Xhosa. (B) Ancestry segments in ‡Khomani. (C) Ancestry segments in Sandawe.
(TIFF)

S7 Fig. (A) Distributions of derived allele frequencies, deletions and duplications in the southern African populations. (B) The number of CNVs per individual in each of the southern African populations.
(TIFF)

S1 Table. Populations that were included in population structure analysis of South African Coloureds (SAC). The southern Bantu-speakers in this study are represented by the Sotho-Tswana (STS) inhabiting the central plateau of southern Africa; the Nguni, represented by Zulu (ZUL), Xhosa (XHS) speakers, inhabiting KwaZulu Natal on the east coast and the Eastern Cape, and the Herero (HER) inhabiting northern Namibia, respectively (S1 Table). The eastern Bantu-speakers are mostly populations inhabiting the central lake regions and the east coast of Africa.
(DOC)

S2 Table. Pairwise population genetic distance.
(DOC)

S3 Table. Three-population tests for 'treeness': The signal of admixture in the southern African populations. Shown are all populations with at least one negative f3 statistic, the names of the putative mixing populations (population1 and 2, not necessarily the populations that

actually mixed historically) that give rise to the minimum f3 statistic, the value of the statistic, and its standard error.
(DOC)

S4 Table. Outlier points distant from the regression line by 0.05 units in southern Africa populations, identified from linear regression of genetic and geographic distance between southern African populations and Bantu-speakers. These outliers show possible obstacles to migration.
(DOC)

S5 Table. Characteristics of haplotype blocks for all chromosomes in each of the analysis panels.
(DOC)

S6 Table. Distribution of the number of recombination hotspots in each population, where recombination rates are identified as regions with a recombination rate greater than 5 times the background chromosomal recombination rate. Values in parentheses indicate the number of recombination hotspots shared with CEU and YRI respectively, for each of the southern African populations included in this study.
(DOC)

S7 Table. Power to recover a recombination hotspot after a population bottleneck when SNPs have been ascertained in (a) the genotyped population and (b) a population that has diverged from the genotyped population τ generations before the present. Power is estimated as the proportion of simulated datasets in which a recombination hotspot is inferred with strength 50 times the background recombination rate, and which lies within 25kb of the simulated hotspot.
(DOC)

S8 Table. Number of monomorphic SNPs, and proportion of SNPs monomorphic with respect to the total number of SNPs shared (n = 798807) between the HapMap and southern African datasets.
(DOC)

S9 Table. Pairwise relatedness (*r*) between individuals and mean per individual homozygosity (*h*) across all SNP loci for each of the study populations. Values in parentheses are standard errors.
(DOC)

S1 Text. Haplotype phasing, linkage disequilibrium and imputation.
(DOC)

S2 Text. Simulations.
(DOC)

S3 Text. Imputation of missing data.
(DOC)

S4 Text. Fine Scale recombination mapping.
(DOC)

S5 Text. Genotype and copy number calling.
(DOC)

S6 Text. Data filtering.
(DOC)

Acknowledgments

We would like to acknowledge all participants who have volunteered to participate in our research, Affymetrix, Dr. Reinhard Hiller (CPGR), Geoffrey Yang (Affymetrix) and Izak van Niekerk (Southern Cross Biotechnology) for their roles in ensuring the genotyping of the southern African individuals in this study, Dr. Wayne Delport for his contribution to the study, Dr. Jonathan Marchini and Chris Spencer (Oxford University), for discussion of the concepts presented in this paper, Dr. George Rebello and Dr. Victoria Nembaware for discussion and proof-reading, Dr. Debo Adeyemo and Dr. Charles Rotimi for comments on a draft manuscript, and The Meraka Institute (http://www.meraka.org.za/) for computational resources.

Author Contributions

Conceived and designed the experiments: RR HS. Performed the experiments: RR HS. Analyzed the data: ERC AM MT NM CS. Contributed reagents/materials/analysis tools: CS NM. Wrote the paper: ERC HS RR.

References

1. Frazer KA, Ballinger DG, Cox DR, Hinds DA, Stuve LL, et al. (2007) A second generation human haplotype map of over 3.1 million SNPs. Nature 449: 851–861. PMID: 17943122
2. Jakobsson M, Scholz SW, Scheet P, Gibbs JR, Vanliere JM, et al. (2008) Genotype, haplotype and copy-number variation in worldwide human populations. Nature 451: 998–1003. doi: 10.1038/nature06742 PMID: 18288195
3. Li JZ, Absher DM, Tang H, Southwick AM, Casto AM, et al. (2008) Worldwide Human Relationships Inferred from Genome-Wide Patterns of Variation. Science 319: 1100–1104. doi: 10.1126/science.1153717 PMID: 18292342
4. McVean G, Spencer CCA, Chaix R (2005) Perspectives on Human Genetic Variation from the HapMap Project. PLoS Genetics 1: e54. PMID: 16254603
5. The International HapMap C (2005) A haplotype map of the human genome. Nature 437: 1299–1320. PMID: 16255080
6. Cann HM, de Toma C, Cazes L, Legrand M-F, Morel V, et al. (2002) A Human Genome Diversity Cell Line Panel. Science 296: 261b–262.5. PMID: 11954565
7. Sabeti PC, Varilly P, Fry B, Lohmueller J, Hostetter E, et al. (2007) Genome-wide detection and characterization of positive selection in human populations. Nature 449: 913–918. PMID: 17943131
8. Tang K, Thornton KR, Stoneking M (2007) A new Approach for Using Genome Scans to Detect Recent Positive Selection in the Human Genome. PLoS Biology 5: e171. PMID: 17579516
9. McVean GAT, Myers SR, Hunt S, Deloukas P, Bentley DR, et al. (2004) The fine- scale structure of recombination rate variation in the human genome. Science 304: 581–584. PMID: 15105499
10. Voight BF, Kudaravalli S, Wen X, Pritchard JK (2006) A map of Recent Positive Selection in the Human Genome. PLoS Biology 4: e72. PMID: 16494531
11. Williamson S, Hubisz MJ, Clark AG, Payseur BA, Bustamante CD, et al. (2007) Localizing recent adaptive evolution in the human genome. PLoS Genetics 3: e90. PMID: 17542651
12. Stringer CB, Andrews P (1988) Genetic and fossil evidence for the origin of modern humans. Science 239: 1263–1268. PMID: 3125610
13. Conrad DF, Jakobsson M, Coop G, Wen X, Wall JD, et al. (2006) A worldwide survey of haplotype variation and linkage disequilibrium in the human genome. Nat Genet 38: 1251–1260. PMID: 17057719
14. Tishkoff SA, Williams SM (2002) Genetic analysis of African populations: human evolution and complex disease. Nat Rev Genet 3: 611–621. PMID: 12154384
15. Tishkoff SA, Kidd KK (2004) Implications of biogeography of human populations for 'race' and medicine. Nat Genet 36: S21–27. PMID: 15507999

16. Pickrell JK, Patterson N, Loh P-R, Lipson M, Berger B, et al. (2014). Ancient west Eurasian ancestry in southern and eastern Africa. Proc Natl Acad Sci: 111(7):2632–7. doi: 10.1073/pnas.1313787111 PMID: 24550290

17. Behar DM, Villems R, Soodyall H, Blue-Smith J, Pereira L, et al. (2008) The dawn of human matrilineal diversity. Am J Hum Genet 82: 1130–1140. doi: 10.1016/j.ajhg.2008.04.002 PMID: 18439549

18. Campbell MC, Tishkoff SA (2008) African genetic diversity: implications for human demographic history, modern human origins, and complex disease mapping. Annu Rev Genomics Hum Genet 9: 403–433. doi: 10.1146/annurev.genom.9.081307.164258 PMID: 18593304

19. Gonder MK, Mortensen HM, Reed FA, de Sousa A, Tishkoff SA (2007) Whole-mtDNA genome sequence analysis of ancient African lineages. Mol Biol Evol 24: 757–768. PMID: 17194802

20. Tishkoff SA, Gonder MK, Henn BM, Mortensen H, Knight A, et al. (2007) History of Click-Speaking Populations of Africa Inferred from mtDNA and Y Chromosome Genetic Variation. Mol Biol Evol 24: 2180–2195. PMID: 17656633

21. Knight A, Underhill PA, Mortensen HM, Zhivotovsky LA, Lin AA, et al. (2003) African Y chromosome and mtDNA divergence provides insight into the history of click languages. Curr Biol 13: 464–473. PMID: 12646128

22. Wood ET, Stover DA, Ehret C, Destro-Bisol G, Spedini G, et al. (2005) Contrasting patterns of Y chromosome and mtDNA variation in Africa: evidence for sex-biased demographic processes. Eur J Hum Genet 13: 867–876. PMID: 15856073

23. Watkins WS, Rogers AR, Ostler CT, Wooding S, Bamshad MJ, et al. (2003) Genetic variation among world populations: inferences from 100 Alu insertion polymorphisms. Genome Res 13: 1607–1618. PMID: 12805277

24. Ehret C (2001) An African classical age: Eastern and southern Africa in world history, 1000 B.C. to A.D. 400. Int. J. of Afric. Hist. Stud: 34, 667–669.

25. Ehret C, Posnansky M (1982).The archaeological and Linguistic Reconstruction of African History. California: University of California Press. 211–221p.

26. Henn BM, Gignoux CR, Jobin M, Granka JM, Macpherson JM, et al. (2011). Hunter-gatherer genomic diversity suggests a southern African origin for modern humans. PNAS 108:5154–5162. available: http://www.pnas.org/content/108/13/5154.full. Accessed February 3, 2011. doi: 10.1073/pnas.1017511108 PMID: 21383195

27. Ehret C (1971) Southern Nilotic History: Linguistic Approaches to the Study of the Past. Chicago: Northwestern Univ. Press. 112–127 p.

28. Ehret C. (1974) Ethiopians and east Africans: The problem of Contacts. Nairobi: east African Publishing House Press.

29. Nurse GT, Weiner JS, Jenkins T (1985) The peoples of southern Africa and their affinities. New York: Oxford University Press. 209–256 p.

30. Soodyall H, Makkan H, Haycock P, Naidoo T (2008) The genetic prehistory of the Khoe and San. Southern African Humanities Khoekhoe and the earliest herders in southern Africa. pp. 37–48.

31. Jenkins T, Zoutendyk A, Steinberg A (1970) Gammaglobulin groups (Gm and Inv) of various southern African populations. Am. J. of Physical Anthropology 32: 197–218. PMID: 4191313

32. Besten MP (2006). Transformation and Reconstitution of Khoe-San Identities: AAS Le Fleur I, Griqua Identities and Post-apartheid Khoe-San Revivalism (1894–2004). Leiden: University of Leiden Press. 85–189 p

33. Lachance J, Vernotm B, Elbers CC, Ferwerda B, Froment A, et al. (2012) Evolutionary history and adaptation from high-coverage whole-genome sequences of diverse African hunter-gatherers. Cell: 150: 457–469. Available: http://www.sciencedirect.com/science/article/pii/S0092867412008318. Accessed 3 August, 2012. doi: 10.1016/j.cell.2012.07.009 PMID: 22840920

34. Elphick R (1985) Khoikhoi and the founding of white South Africa. Johannesburg: Ravan Press.

35. Gurdasani, D, Carstensen T, Tekola-Ayele F, Pagani L, Tachmazidou I, et al. (2014). The African Genome Variation Project shapes medical genetics in Africa. Nature. doi: 10.1038/nature13997

36. Schlebusch CM, Lombard M, Soodyall H. (2013). MtDNA control region variation affirms diversity and deep sub-structure in populations from southern Africa. BMC Evolutionary Biology. 13: 56. doi: 10.1186/1471-2148-13-56 PMID: 23445172

37. Rosenberg NA, Pritchard JK, Weber JL, Cann HM, Kidd KK, et al. (2002) Genetic structure of human populations. Science 298: 2381–2385. PMID: 12493913

38. Tishkoff SA, Reed FA, Friedlaender FR, Ehret C, Ranciaro A, et al. (2009). The genetic structure and history of Africans and African Americans. Science 324:1035–1044 pp.

39. Pickrell JK, Patterson N, Barbieri C, Berthold F, Gerlach L, et al. (2012). The genetic prehistory of southern Africa. Nat. Communications 3:1143. Available:http://www.nature.com/ncomms/journal/v3/n10/full/ncomms2140.html. Accepted 17 September, 2012. doi: 10.1038/ncomms2140 PMID: 23072811

40. Pickrell JK, Pritchard JK (2012) Inference of Population Splits and Mixtures from Genome-Wide Allele Frequency Data. PLoS Genet 8(11): e1002967. doi: 10.1371/journal.pgen.1002967 PMID: 23166502

41. Hellenthal G, Busby J, Band G, Wilson J, Capelli C, et al. (2014) A Genetic Atlas of Human Admixture History. Science 343, 747 (2014); doi: 10.1126/science.1243518 PMID: 24531965

42. Nielsen R, Signorovitch J (2003) Correcting for ascertainment biases when analyzing SNP data: applications to the estimation of linkage disequilibrium. Theoretical Population Biology 63: 245–255. PMID: 12689795

43. Tang H, Choudhry S, Mei R, Morgan M, Rodriguez-Cintron W, et al.(2007) Recent genetic selection in the ancestral admixture of Puerto Ricans. Am. J. Hum. Genet. 81: 626–633. PMID: 17701908

44. GeneCard database (HYPERLINK "www.genecards.org).

45. Chimusa ER, Daya M, Möller M, Ramesar R, Henn BM, et al. (2013) Determining Ancestry Proportions in Complex Admixture Scenarios in South Africa Using a Novel Proxy Ancestry Selection Method. PLoS ONE 8(9): e73971. doi: 10.1371/journal.pone.0073971 PMID: 24066090

46. Baran Y, Pasaniuc B, Sankararaman S, Torgerson DG, Gignoux C, et al. (2012). Fast and accurate inference of local ancestry in Latino populations. Bioinformatics 28(10):1359–67. doi: 10.1093/bioinformatics/bts144 Available:http://bioinformatics.oxfordjournals.org/content/28/10/1359.full. Accessed 11 April, 2012. PMID: 22495753

47. Basu A, Tang H, Zhu X, Gu CC, Hanis C, et al. (2008) Genome- wide distribution of ancestry in Mexican Americans. Hum Genet 124(3):207–14. doi: 10.1007/s00439-008-0541-5 Available:http://www.ncbi.nlm.nih.gov/pmc/articles/PMC3131689/. Accepted August 28, 2008. PMID: 18752003

48. Bryc K, Auton A, Nelson MR, Oksenberg JR, Hauser SL, et al. 2010. Genome-wide patterns of population structure and admixture in west Africans and African Americans. PNAS 107:786–791. Available: http://www.pnas.org/content/107/2/786.full. Accepted January 12, 2010. doi: 10.1073/pnas.0909559107 PMID: 20080753

49. Oleksyk TK, Smith MW, O'Brien SJ. (2010) Genome-wide scans for footprints of natural selection. Philos Trans R Soc Lond B Biol Sci 365: 185–205. doi: 10.1098/rstb.2009.0219 PMID: 20008396

50. Wenfei J, Shuhua X, Haifeng W, Yongguo Y, Yiping S, et al. (2012) Genome- wide detection of natural selection in African Americans pre- and post-admixture. Genome Res 22: 519–527. doi: 10.1101/gr.124784.111 PMID: 22128132

51. Herman JP, Jullien N, Guillen S, Enjalbert A, Pellegrini I, et al. (2012) Research resource: A genome-wide study identifies potential new target genes for POU1F1. Mol. Endocrinol 26(8):1455–1463p. Available: http://dx.doi.org/10.1210/me.2011-130. Accessed 25 May 25 2012. doi: 10.1210/me.2012-1209 PMID: 22844064

52. Inoue H, Mukai T, Sakamoto Y, Kimura C, Kangawa N, et al. (2012) Identification of a novel mutation in the exon 2 splice donor site of the POU1F1/PIT-1 gene in Japanese identical twins with mild combined pituitary hormone deficiency. Clin Endocrinol (Oxf) 76:78–87. doi: 10.1111/j.1365-2265.2011.04165 PMID: 21722153

53. Rappaport N, Nativ N, Stelzer G, Twik M, Guan-Golan Y, et al. (2013) MalaCards: an integrated compendium for diseases and their annotation. Database (Oxford) 2013: bat018. Available: http://www.ncbi.nlm.nih.gov/pmc/articles/PMC3625956/. Accessed 12 April 12 2013.

54. Korn JM, Kuruvilla FG, McCarroll SA, Wysoker A, Nemesh J, et al. (2009). Integrated genotype calling and association analysis of SNPs, common copy number polymorphisms and rare CNVs. Nat. Genet. 40(10):1253–1260p. Available: http://www.ncbi.nlm.nih.gov/pmc/articles/PMC2756534/. Accessed 4 October 2009.

55. McCarroll SA, Kuruvilla FG, Korn JM, Cawley S, Nemesh J, Wysoker A, et a. (2008) Integrated detection and population-genetic analysis of SNPs and copy number variation. Nat. Genet. 40: 1166–1174p.

56. Zhang J, Feuk L, Duggan GE, Khaja R, Scherer SW (2006) Development of bioinformatics resources for display and analysis of copy number and other structural variants in the human genome. Cytogenet Genome Res. 115: 205–214. PMID: 17124402

57. Redon R, Ishikawa S, Fitch KR, Feuk L, Perry GH, et al. (2006) Global variation in copy number in the human genome. Nat. 444: 444–454.

58. Conrad DF, Andrews TD, Carter NP, Hurles ME, Pritchard JK (2006) A high- resolution survey of deletion polymorphism in the human genome. Nat Genet 38: 75–81. PMID: 16327808

59. Sabeti PC, Schaffner SF, Fry B, Lohmueller J, Varilly P, et al. (2006) Positive natural selection in the human lineage. Science 312: 1614–1620. PMID: 16778047

60. Price AL, Helgason A, Palsson S, Stefansson H, St. Clair D, et al. (2009) The Impact of Divergence Time on the Nature of Population Structure: An Example from Iceland. PLoS Genet 5(6): e1000505. doi: 10.1371/journal.pgen.1000505 PMID: 19503599

61. Bhatia G, Tandon A, Patterson N, Aldrich MC, Ambrosone CB, et al. (2014) Genome-wide scan of 29,141 African Americans finds no evidence of directional selection since admixture. Am. J. Hum Genet 95(4):437–44. Available: http://dx.doi.org/10.1016/j.ajhg.2014.08.011. doi: 10.1016/j.ajhg.2014.08.011 PMID: 25242497

62. Seldin MF, Pasaniuc B, Price AL. (2011). New approaches to disease mapping in admixed populations. Nat. Rev. Genet. 12(8):523–8. doi: 10.1038/nrg3002 PMID: 21709689

63. Bhatia G, Patterson N, Sankararaman S, Price A. L. (2013). Estimating and interpreting FST: The impact of rare variants. Genome research, 23(9), 1514–1521. doi: 10.1101/gr.154831.113 PMID: 23861382

64. Adeyemo A, Rotimi C. (2014) What does genomic medicine mean for diverse populations? Mol Genet Genomic Med. 2(1): 3–6 doi: 10.1002/mgg3.63 PMID: 24498625

65. University of Cape Town, Private Bag X3, Rondebosch 7701, South Africa.

66. University of Witwatersrand, 1 Jan Smuts Avenue Braamfontein 2000 Johannesburg, South Africa.

67. Glaubitz JC, Rhodes E, Dewoody A (2003) Prospects for inferring pairwise relationships with single nucleotide polymorphisms. Molecular Ecology 12: 1039–1047pp.

68. Patterson N, Price AL, Reich D (2006) Population structure and eigenanalysis. PLoS Genet. 2(12): e190. PMCID: PMC1713260. PMID: 17194218

69. Alexander DH, Novembre J., Lange K. Fast model-based estimation of ancestry in unrelated individuals. Genome Research, 19:1655–1664. doi: 10.1101/gr.094052.109 PMID: 19648217

70. Scheet P, Stephens M (2006) A Fast and flexible statistical model for large-scale population genotype data: Applications to inferring missing genotypes and haplotypic phase. Am. J. Hum. Genet. 78: 629–644pp.
